# Ballistic Energy Transport via Long Alkyl Chains: A New Initiation Mechanism

**DOI:** 10.1021/acs.jpcb.4c03386

**Published:** 2024-09-02

**Authors:** Sithara U. Nawagamuwage, Elliot S. Williams, Md Muhaiminul Islam, Igor V. Parshin, Alexander L. Burin, Nathalie Busschaert, Igor V. Rubtsov

**Affiliations:** Department of Chemistry, Tulane University, New Orleans, Louisiana 70118, United States

## Abstract

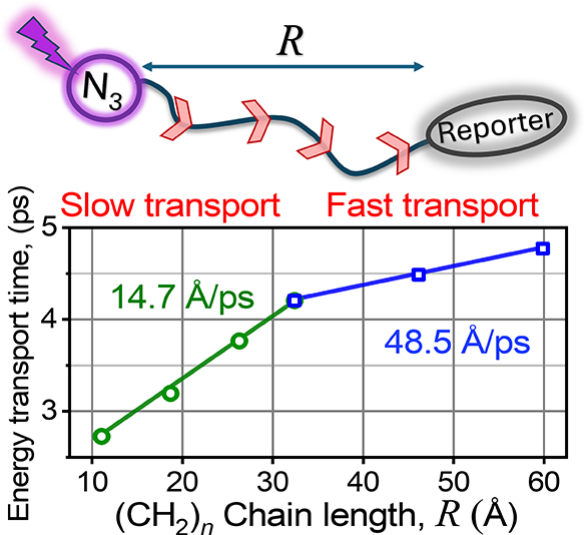

In an effort to increase the speed and efficiency of ballistic energy transport via oligomeric chains, we performed measurements of the transport in compounds featuring long alkyl chains of up to 37 methylene units. Compounds of the N_3_-(CH_2_)_*n*_-COOMe type (denoted as az*n*ME) were synthesized with *n* = 5, 10, 15, 19, 28, 37 and studied using relaxation-assisted two-dimensional infrared spectroscopy. The speed of the ballistic transport, initiated by the N_3_ tag excitation, increased ca. 3-fold for the longer chains (*n* = 19–37) compared to the shorter chains, from 14.7 to 48 Å/ps, in line with an earlier prediction (Nawagamuwage et al. 2021, *J. Phys. Chem. B*, **125**, 7546). Modeling, based on solving numerically the Liouville equation, was capable of reproducing the experimental data only if three wavepackets are included, involving CH_2_ twisting (Tw), wagging (W), and rocking (Ro) chain bands. The approaches for designing molecular systems featuring a higher speed and efficiency of energy transport are discussed.

## 1 Introduction

Vibrational energy flow in molecules has been a topic of interest in chemistry since Arrhenius’s explanation of heat dependence of reaction rates.^1,2^ Even though in nature the most common form of heat transfer involves diffusive hops of vibrational energy by Brownian motion, there is the possibility of transferring vibrational energy ballistically in condensed matter. This requires strong coupling of a number of local states extended over a significant range of distances forming delocalized states.^3−11^ Particularly, it was shown recently that vibrational energy delivery controls enzyme functioning in soybean lipoxygenase^12^ and intersystem crossing rate in iron(II)-tris(bipyridine).^13^ The presence of strong covalent bonds in periodic molecular chains results in generally strong interactions of nearest neighbor local sites, facilitating long-range delocalization. Such delocalized coupled states are traditionally described by dispersion relations, ω(*q*), where ω is the frequency (energy) of the coupled states and *q* is its wavevector.^8,14^ Acoustic phonons in materials and molecular chains are responsible for heat conduction.^15,16^ Optical phonons can be used to transfer larger quanta of energy, released, e.g., in chemical reactions, ballistically to longer distances, making such systems potentially useful as new materials for heat management, molecular electronics, and sensors.^17−20^ Recent work^21,22^ suggests similar applications of vibrational polaritons which are quantum superpositions of vibration and infrared light.

**Figure 1:**
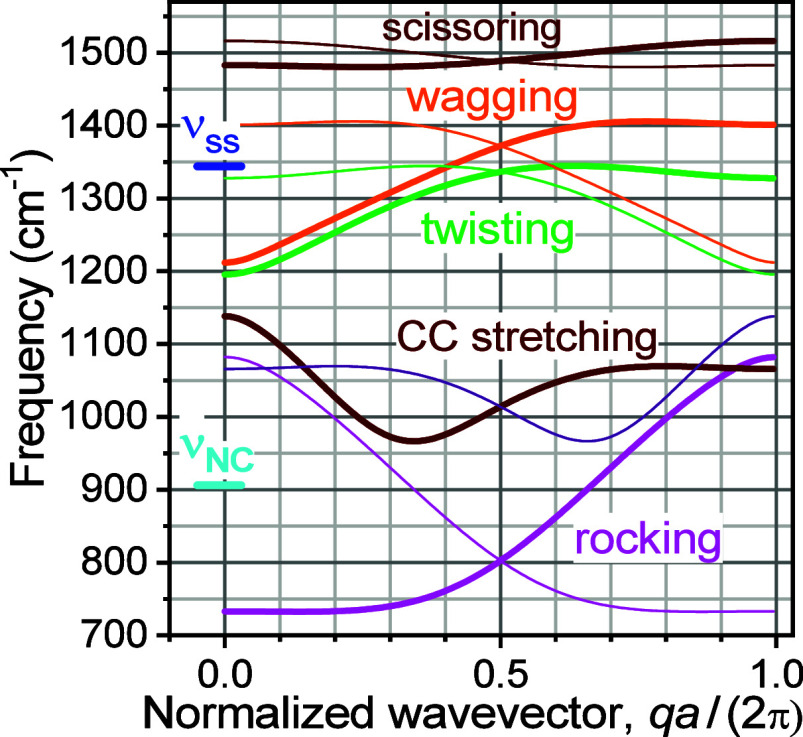
Dispersion curves for five optical bands of linear alkyl chains. Vibrational frequencies of the azido moiety of the N_3_-alkyl compound, the N_3_ asymmetric stretch (ν_ss_) and ν_NC_, are also shown (reproduced from ref (23). Copyright 2015, American Chemical Society).

Dispersion curves for infinitely long linear alkyl chains involving CH_2_ scissoring, wagging (W), twisting (Tw), and rocking (Ro) bands and a C–C stretching band are shown in Figure 1. The widths of different bands and the shapes of the dispersion curves differ as interaction strengths and signs of the local vibrational states of different types vary. Actual energies of coupled states of a band for a chain of finite length can be obtained from its dispersion curve. By exciting a superposition of two or more delocalized chain states, a vibrational wavepacket is formed.

Once formed, the wavepacket propagates in the chain with time, moving its energy along the chain at a nearly constant speed. Such energy transport is called ballistic transport; it is typically much faster and more efficient than diffusive transport.^14^ The transport speed of the wavepacket formed in the vicinity of wave vector *q*_0_ (its group velocity at *q*_0_) is determined by the slope of the dispersion curve: *V*(*q*_0_) = (*∂*ω/*∂q*)|_*q*=*q*_0__. Depending on how wide the range of the wavevectors involved in the wavepacket is, the wavepacket transport speed can vary significantly. Therefore, understanding the process of formation of the wavepacket in the chain is of ultimate importance for describing the energy transport in such chains.

Ballistic energy transport via covalent bonds in molecules has been studied actively over the past two decades. The transport was initiated by electronic excitation (∼2 eV)^24,25^ or by vibrational excitation of an end group of oligomeric chains (1500–2100 cm^–1^).^26^ Various chain types were investigated following vibrational excitation and detection using relaxation-assisted two-dimensional infrared (RA 2DIR) spectroscopy.^27,28^ In these experiments, the energy transport was initiated by exciting a high-frequency mode of a chain end group (the tag). Vibrational relaxation of the excited mode initiates a wavepacket, which propagates freely in the chain. The arrival of the wavepacket to another end of the chain is recorded via 2DIR as a change in the frequency of the reporter mode located at this end of the chain caused by excess energy arrival. The transport speed is measured as an inverse slope of the dependence of the energy arrival time as a function of the chain length.

The transport speed was found to be dependent on the chain type (PEG,^26,29−31^ perfluoroalkyl,^32^ alkyl,^23,33,34^ and oligo-*p*-phenylene^35^) and on the selection of the tag mode initiating the transport but not on the reporter mode selection. For example, a speed of 80 Å/ps (8 km/s) was observed in oligophenylene chains,^35^ which is ca. 5-times larger than the speed of sound in water. The transport speed via linear alkyl chains up to 15 methylene units, measured using RA 2DIR, was found to be strongly dependent on the type of the end-group initiating the transport with 14.4, 8.0, and 4.3 Å/ps for the N_3_ (2100 cm^–1^), NHS-ester (1740 cm^–1^), and amide (1640 cm^–1^) mode initiations, respectively, associated with transport via different optical bands of the chain.^33^

**Figure 2:**
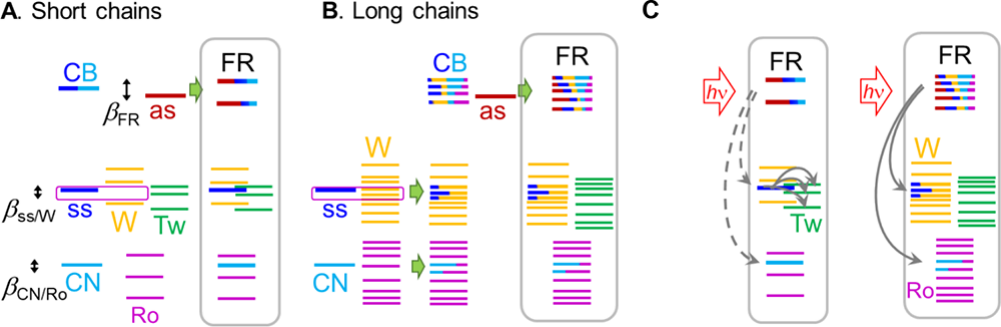
(A, B) Energy schematics show harmonic mixing of the end-group and chain states and nonlinear mixing of ν_as_ with the combination band (CB) of predominantly ν_ss_ and ν_CN_ for the cases of short (A) and long (B) chains. The interaction energies, β_ss/W_ ∼ 10 cm^–1^, β_CN/Ro_ ∼ 9 cm^–1^, and β_FR_ ∼ 30 cm^–1^, are indicated with double-sided arrows (β_ss/Tw_ < 0.1 cm^–1^). Formation of the harmonically mixed end-group and chain states is shown in the single-quantum manifold within gray boxes. The resulted combination bands involving ν_ss_ and ν_CN_ local models are labeled as CB. The nonlinear mixture of the CB with ν_as_ results in the coupled two quanta states denoted as FR. The IR intensities of these states (GS + *h*ν → FR) scale as the square of the contribution of ν_ss_ in the coupled state wavefunction. (C) Mechanisms of wavepacket formation for short (left) and long (right) chains.

A recent comprehensive study on the wavepacket initiation mechanisms using azido groups in alkyl chains of length up to 15 repeating units identified several mechanisms of wavepacket initiation.^36^ It was shown that the asymmetric N≡N stretching mode (ν_as_, 2100 cm^–1^, the tag) relaxes predominantly into a combination band of the symmetric N_3_ stretching mode, ν_ss_, and N–C stretching mode, ν_NC_ (Figures 1 and 2).^23,37^ A fast intramolecular vibrational energy redistribution (IVR) process of ν_ss_ populates a range of Tw chain states as well a low frequency mode, creating a wavepacket in the Tw band (Figure 2A,C, left), which is the dominant mechanism of wavepacket formation for short chains. It was predicted that for longer chains (*n* > 15), additional mechanisms of wavepacket formation can become efficient, resulting in wavepackets in the W and Ro bands. The new wavepacket formation channels are expected to become efficient at increased chain-state density so that more than one W chain state can be found within ν_ss_ (*n* > 15) and more than one Ro state is found within ν_CN_ (*n* > 25), Figure 2C, right.

Here, we report on RA 2DIR studies (Section 3.1) and density matrix modeling (Section 3.2) of the energy transport in molecular wires of the ^14^N_3_(CH_2_)_*n*_-COOMe type (denoted as az*n*ME) with *n* = 5, 10, 15, 19, 28, and 37. The discussion section (Section 4) describes the possible steps to increase the speed, efficiency of initiation, and efficiency of ballistic transport.

## 2 Experimental Details

### 2.1 Synthesis

**Scheme 1:**
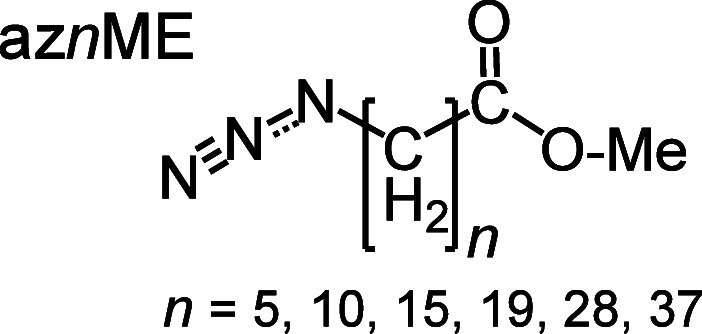
Structure of the az*n*ME Compounds

A series of compounds featuring linear alkyl chains of lengths *n* = 5, 10, 15, 19, 28, and 37 each terminated with one azido and one methyl ester end groups, denoted as az*n*ME, were synthesized (Scheme 1). For *n* = 5, 10, and 15, the compounds could be easily obtained using a substitution reaction from commercially available bromo-terminated carboxylic acids with NaN_3_ followed by esterification to methyl esters. The longer chains (*n* = 19, 28, and 37) had to be synthesized in a stepwise fashion, using alkene cross-metathesis to elongate the chains. Thus, az19ME was obtained from the cross-metathesis of a C11 carboxylate ester containing a terminal alkene and 11-bromoundecene followed by a reduction of the internal alkene and substitution of the terminal bromine with azide. Similarly, az28ME was obtained from the cross-metathesis of one of the az19ME intermediates with 11-bromoundecene followed by a reduction of the internal alkene and substitution of the terminal bromine with azide while az37ME was obtained from the cross-metathesis of one of the az28ME intermediates with 11-bromoundecene followed by a reduction of the internal alkene and substitution of the terminal bromine with azide. A detailed reaction scheme, reaction conditions, and characterization are given in the SI.

### 2.2 Sample Preparation

For FTIR and 2DIR measurements, each compound was dissolved in CDCl_3_ to prepare a 30 mM solution. All experiments were performed in a sample cell with a 100 μm Teflon spacer separating two 1 mm-thick CaF_2_ windows.

### 2.3 2DIR Measurements

We used relaxation-assisted two-dimensional infrared (RA 2DIR) spectroscopy^38^ to study energy transport along alkyl chains. A comprehensive description of the fully automated dual frequency three-pulse photon echo 2DIR spectrometer used is formerly published elsewhere.^39^ Briefly, an output of 800 nm wavelength from a Ti:sapphire laser producing 1.5 W power at 1 kHz repetition rate with 80 fs pulse duration (Libra, Coherent) was passed through a dual optical parametric amplifier (OPA, Palitra-duo, Quantronix) and a pair of difference frequency generation (DFG) units (NIR Quantronix) to generate tunable mid-IR pulses in the frequency range from 500 to 5000 cm^–1^, featuring pulse energy ranging from 1.0 to 10 μJ. The pulse energies of the mid-IR beams at the sample were 1.5 and 1 μJ at 2100 and 1730 cm^–1^, respectively.

The instrument is equipped with a mid-IR beam direction stabilization schematic^40^ and a schematic for setting the phase-matching geometry for mid-IR beams at the sample^39^ to enable automated navigation to any cross or diagonal 2DIR peak. The spectral width of the mid-IR pulses was ∼150 cm^–1^, and the instrument response function was ∼180 fs.

The 2DIR measurements were performed by scanning the delay between the first two mid-IR pump pulses, (τ) originating from the same DFG unit, at a fixed waiting time (*T*), which is the delay between the second and third pulses, and recording the heterodyned spectrum in the frequency range of interest (λ → ω_*t*_) for every *T*. Fourier transformation along τ results in the ω_τ_ axis in the 2DIR spectrum, shown as the ordinate. A typical 2DIR spectrum contained ∼250 points along the τ direction, which took 1–3 min to acquire. For RA 2DIR measurements, the waiting time, *T*, was scanned with nonconstant delay steps ranging from 100 fs at small waiting times to 5 ps at large waiting times. Typical waiting-time dependences contained 40–50 points along *T*, which took ca. 1.5 h to acquire.

### 2.4 Theoretical Modeling

We described the transport by solving Liouville–Bloch equations for the density matrix as in ref (41). The hopping through the chain of *n* sites, enumerated by numbers 1, 2, ..., *n*, was characterized by nearest neighbor and next neighbor couplings, β and γ, respectively, as introduced below in eq 1, and dephasing and relaxation rates, *k*_deph_ and *k*_rlx_. The first site of the chain is coupled to the initiation zeroth site, characterized by energy *E*_0_, with coupling strength β_tag_. The last site, *n*, is coupled to the reporter, whose population obeys the kinetic equation d*P*/d*t* = *k*_rep_ρ_*nn*_ – *P*/*T*_c_, where *P* is the population of the reporter, *k*_rep_ is the rate of the energy transfer from the *n*th site of the chain to the reporter state, ρ_*nn*_ is the diagonal element of the density matrix, characterizing the excitation density at the site adjacent to the reporter and *T*_c_ is the reporter cooling rate to the solvent. We do not assume that the reporter C=O stretching mode is populated because its frequency exceeds those of the optical bands responsible for the energy transport. There are several lower frequency modes at the reporter end-group coupled anharmonically to the reporter mode.^42^ Excitation of those modes shifts the reporter mode energy. We assume that the cross-peak intensity is defined by the population of the reporter *P*(*t*); the energy transport time was evaluated as time where the *P*(*t*) trace reaches its maximum. Note that the *T*_c_ time should not be understood as a lifetime of a single mode but as a parameter describing the cooling process of the reporter site to the solvent. The initial conditions were set similarly to that in ref (41) as ρ_ab_(0) = δ_a0_ δ_b0_, where δ_ab_ is a Kronecker symbol.

For transport involving several chain bands, contributions from individual bands were added with appropriate weight factors, as described in Section 3. We consider harmonic initiation of the wavepacket. In the case of its initiation by means of anharmonic transition, the wavepacket is formed if the initiation emerges at a sufficiently short time scale,^43^ as shown for wavepacket initiation in the Tw band.^36^ The present consideration is still relevant even for the anharmonic initiation since the most important observations are associated with the wavepacket transport rather than its initiation.

## 3 Results

### 3.1 RA 2DIR Measurements of the Energy Transport in Alkyl Chains, Initiated by ν_N≡N_

**Figure 3:**
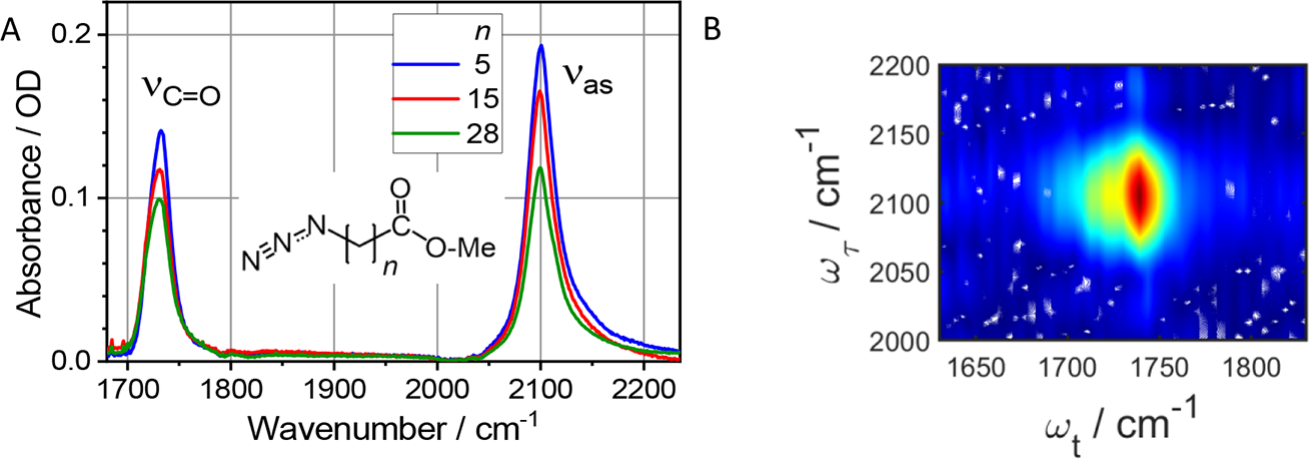
(A) Linear spectra of az*n*ME (*n* = 5, 15, 28) compounds in CDCl_3_. ν_as_ values of the N_3_ moiety and ν_C=O_ of the methyl ester moiety are indicated. (B) 2DIR rephasing spectrum of az28Me at *T* = 5.5 ps focusing at the ν_N≡N_/ν_C=O_ cross peak.

In the set of experiments, the energy transport was initiated by exciting an asymmetric stretch of the azido group at 2100 cm^–1^ (ν_N≡N_, the tag) at one end of the molecule (Figure 3A). The excess energy, introduced to the azido group, relaxes to the alkyl chain, resulting in wavepacket formation. The wavepacket propagates through the chain and arrives at the other end of the molecule, wherein it is observed by the carbonyl stretch of the methyl ester group (ν_C=O_, the reporter) at 1730 cm^–1^ (Figure 3A).

**Figure 4:**
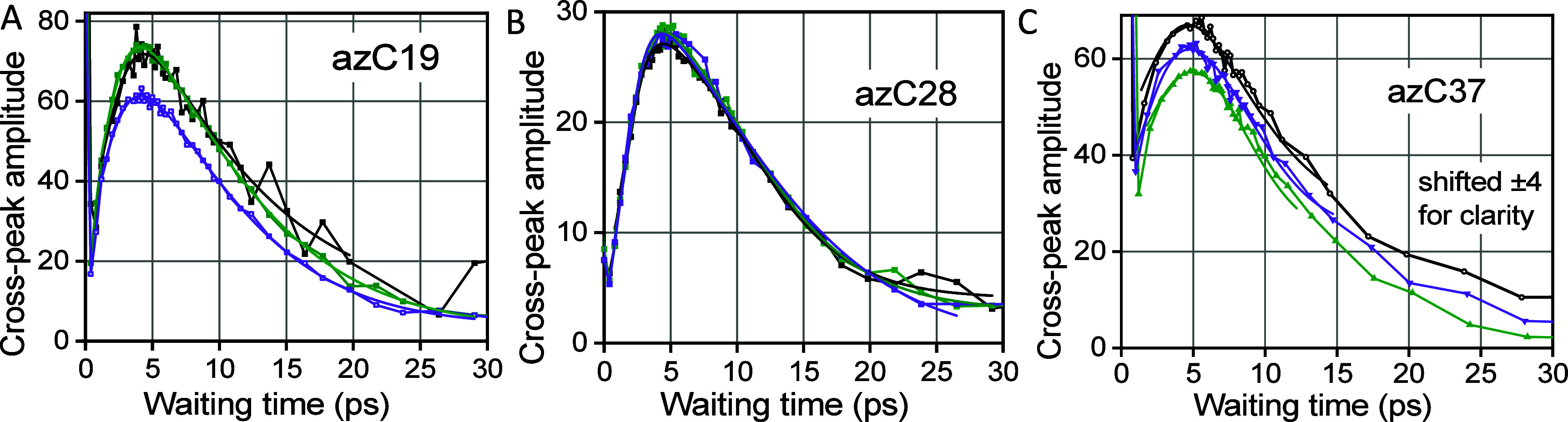
Waiting time traces for the ν_N≡N_/ν_C=O_ cross-peak for (A) az19ME, (B) az28ME, and (C) az37ME. Three traces are shown for each compound. The fit curves with an asymmetric double–sigmoidal function (see Figure S31 in SI), are shown with matching colors. Note that the signal around zero waiting time is caused by a coherent artifact; it decays with the instrument response time.

**Figure 5:**
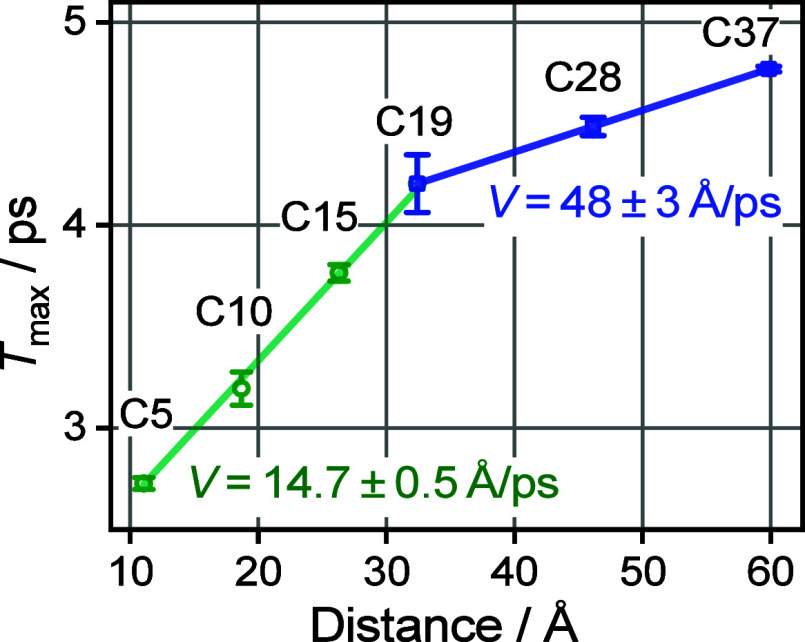
Dependence of *T*_max_ on the tag reporter distance. Linear fits for the az5ME–az19ME and az19ME–az37ME are shown; the resulting energy transport speeds are shown as inset with matching colors.

The energy arrival to the methyl ester moiety is observed as an increase in amplitude of the cross peak between the tag and reporter (Figure 3B). The amplitude of the ν_N≡N_/ν_C=O_ cross-peak in the 2DIR spectrum increases with the waiting time due to the arrival of excess energy to the reporter site (Figure 4). For each waiting time, the ν_N≡N_/ν_C=O_ cross-peak area was integrated and plotted as a function of the waiting time. The resulting waiting time traces are shown in Figure 4 for three indicated compounds. The traces were fitted with an asymmetric double–sigmoidal function (Figure S31 caption) and the curve maximum was determined from the fit. The time taken to reach the highest cross-peak amplitude is termed an energy transport time, *T*_max_. Note that no less than three experiments were performed for each sample (Figure 4) and the reported *T*_max_ value for each sample was obtained by averaging those results. Measurements at different concentrations of the guest resulted in the same *T*_max_ values (Figure S33). The averaged *T*_max_ values were plotted as a function of the tag-reporter distance (Figure 5). Finally, the energy transport speed can be determined as the inverse slope of the *T*_max_(*R*) dependence.

As apparent from Figure 5, the *T*_max_(*R*) dependence is not linear over the whole range of chain lengths but shows a clear kink at *R* ∼ 32 Å. The experimental speed obtained from the linear fit of the data for short chains (*n* < 19) is found at 14.7 ± 0.3 Å/ps, which accords with the previous studies with different reporter groups, such as NHS ester (14.7 ± 0.3 Å/ps),^23^ carboxylic acid (14.4 ± 2 Å/ps),^36^ and an amide (14.0 ± 0.6 Å/ps for amide-I and 13.9 ± 0.6 Å/ps for amide-II).^34^ While the speeds recorded with different reporters were the same, the *T*_max_ values for the chains of the same lengths were different for different reporters, so the whole series in this study was performed with the same reporter: methyl ester. A linear fit of the points for the longer chains (*n* ≥ 19), resulted in the transport speed of 48 ± 3 Å/ps, which is ca. 3 times faster than that for the short chains, suggesting that the mechanism of the transport changes for longer chains.

### 3.2 Modeling of the Wavepacket Transport

A model based on solving the Liouville–von Newman equation for the density matrix was developed, coded in MATLAB, and numerically solved. The az*n*ME chain was represented by *n* local states interacting with each other via nearest, β, and next nearest, γ, neighbor interaction coupling constants. The tag end group was represented by a single state coupled to the nearest chain state, 1, with β_tag_. The numerical solution of the density matrix equation results in the time evolution of the diagonal density matrix elements, which represent excess energy at different sites. The reporter site is added in the ad hoc fashion as a relaxation channel from the last site of the chain with a rate constant, *k*_report_.

The parameters of the chain band were taken from the fit of the computed chain states for infinitely long chain data with an analytical function given by eq 1.^36,44^
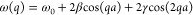
1Here, ω_0_ is the site energy, *a* is the chain period, and *q* is the wavevector of the associated delocalized normal mode.

Previous studies suggested that for the compounds with short chains (*n* ≤ 15), the wavepacket at the CH_2_ twisting (Tw) band dominated the transport.^23^ However, for longer chains, formation of the wavepacket at CH_2_ wagging (W) band is expected, which would result in an increased speed.^36^ Furthermore, a wavepacket at the CH_2_ rocking band (Ro) may contribute for even longer chains (*n* > 30), characterized by a significantly faster speed.

### 3.3 Transport via a Single Chain Band

We first investigated whether the modeling involving a single chain band, Tw, can reproduce the experimental data. We found that while the kink in the *T*_max_(*R*) dependence can be obtained in the calculations, the computed transport speed for the longer chains, limited by the maximum speed supported by the Tw band, is significantly smaller than the experimental speed. The β and γ parameters determine the width of the chain band and thus the maximum group velocity supported by the band (Table 1). We kept β and γ fixed with the values determined from the fit with eq 1 of previously computed dispersion curve for the Tw band (see Table 1).^36^

**Table 1:** Chain Band Parameters for Twisting, Wagging, and Rocking Bands: Site Energy (*E*_0_) and Nearest (β) and Next-Nearest (γ) Coupling Constants^23,36^

chain bands	ω_0_, cm^–1^	β, cm^–1^^a^	γ, cm^–1^^a^	maximal group velocity, Å/ps
wagging	1344	–43	–15	35
twisting	1303	–28	–18	26
rocking	852	–83.8	25.7	69

a The error bars for β and γ, originated from the fit of the dispersion curves with eq 1, were not exceeding 0.5 cm^–1^. However, the computational DFT errors for the dispersion curves are expected to be larger, within ±3 cm^–1^. The error bars for *E*_0_ with respect to the end-group state energies ν_ss,N3_ and ν_C–N_ were estimated to be within 10 cm^–1^.

**Figure 6:**
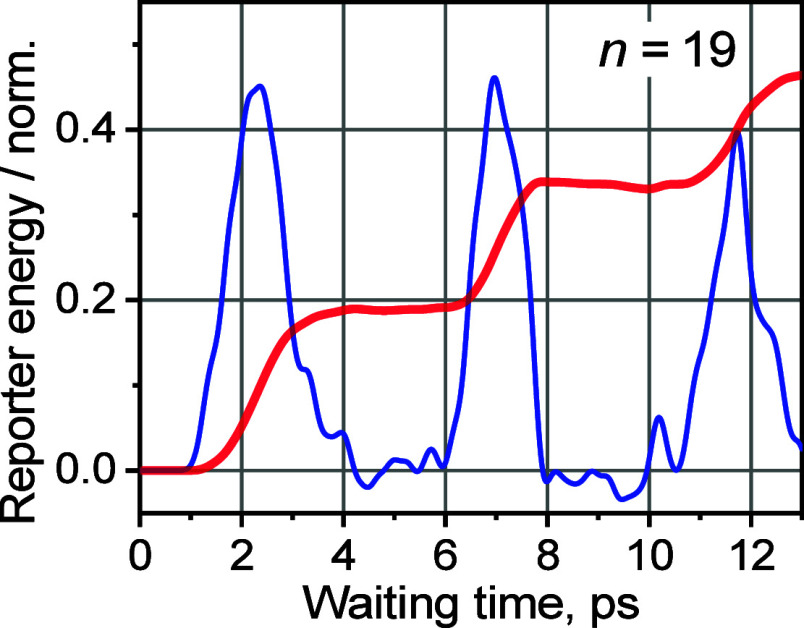
Computed excess energy at the reporter site ((*S*(*T*), red line) transported via the Tw band of the chain with *n* = 19 and its numerical time derivative (blue line) as a function of time when *k*_deph_ = 0 ps^–1^, *k*_rlx_ = 0 ps^–1^, *k*_rep_ = 0.05 ps^–1^, *E*_0_ = −25 cm^–1^, and β_tag_ = 10 cm^–1^.

To clearly observe the wavepacket motion, we first set the modeling parameters so that the wavepacket propagation in the chain occurs with minimal losses inside the chain, as well as small losses to the reporter site. To achieve that, we set the rates of dephasing (*k*_deph_), relaxation (*k*_rlx_), and leak to the reporter (*k*_rep_) to be negligible. The cooling process of the reporter site was also eliminated (large *T*_c_). Under these conditions, the energy at the reporter site, *S*(*T*), accumulates slowly in a step-like fashion (Figure 6, red line). To emphasize the steps in *S*(*T*), the derivative of *S*(*T*), ∂*S*/∂*T*, is shown in Figure 6 with a blue line. The peaks in ∂*S*/∂*T* match the energy leakage bursts from the chain to the reporter site. Notice that the wavepacket undergoes some broadening and deformations due to the nonlinearity of the dispersion relations for optical bands. The repeating peaks feature time separation corresponding to the wavepacket round trip in the chain. Despite the band-state nonlinearity, it is apparent that the wavepacket can potentially exist in the chain for a long time, exceeding 10 ps, given that the dephasing and relaxation of the chain states is slow. It is also clear that the peak in the *S*(*T*) dependence (Figure 4) is formed via a competition of the energy arrival to the reporter site and the reporter site cooling.^45^

**Figure 7:**
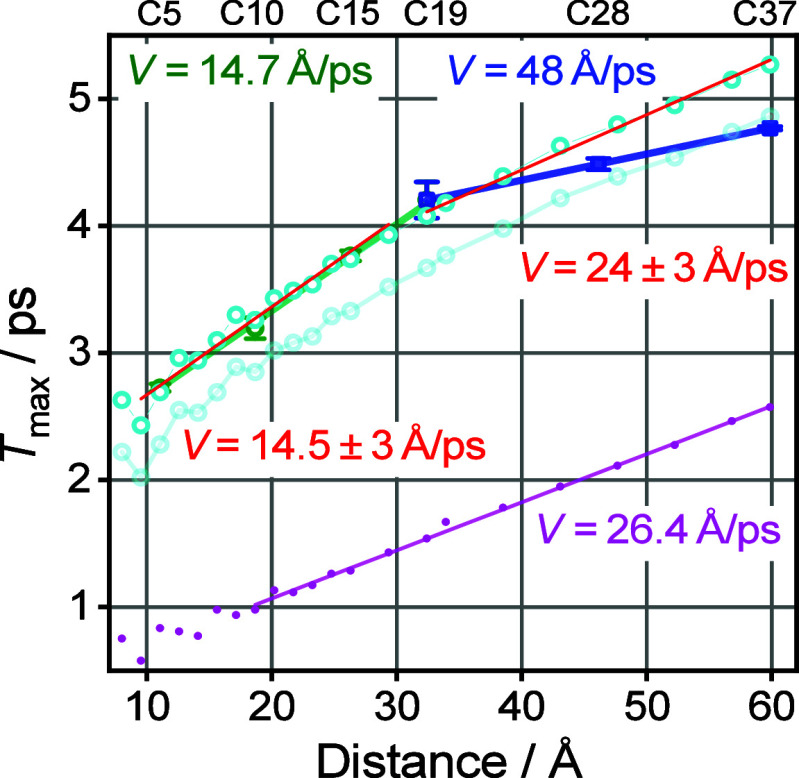
(left) Dependence of *T*_max_ on the tag reporter distance, duplicated from Figure 5 (green and blue symbols and lines), overlaid with the modeling (cyan circles) for different chain lengths. The light cyan circles are the computed values; the data shown with cyan color are shifted up by 0.45 ps, accounting for the time of a wavepacket formation. Linear fits of the modeling data for the *n* of 3–19 and *n* of 19–37 are shown with thin red lines and respective speeds are indicated as insets in red. The modeling parameters were as follows: *E*_0_ = −25 cm^–1^, β_tag_ = 10 cm^–1^, *k*_deph_ = 0.27 ps^–1^, *k*_rlx_ = 0.5 ps^–1^, *k*_rep_ = 4 ps^–1^, and *T*_c_ = 10 ps. The maximum energy flow to the reporter, determined as the maximum of ∂*S*/∂*T*, is shown with magenta dots and its linear fit with magenta line. (right) Experimental waiting time dependence for az19ME (blue) and modeled dependence for *n* = 19 (green). To reduce the fluctuations in the *T*_max_ values, we incorporated inhomogeneity for the energies of the tag state of 10 cm^–1^ (see Section S2 in the SI for more details).

By using realistic parameters for the wavepacket motion, a good match of the modeling and the experiment was achieved but only for short chains (*n* = 3–19), Figure 7. The modeling parameters used are shown in Figure 7 caption. The modeling describes well the presence and the location of the kink in the *T*_max_(*R*) dependence, while the speed for longer chains is not well described.

The *T*_max_ value of the kink in the *T*_max_(*R*) dependence of ca. 3.5 ps dictates the selection of the values for *k*_deph_ and *k*_rlx_ of about (2–3 ps)^−1^. Under these conditions, additional energy round-trips in the chain, caused by the wavepacket reflection at the chain end and then the second reflection at the tag site, became inefficient due to dephasing. Even for a short chain of *n* = 5, the second wavepacket arrival after a roundtrip occurs at ca. 4 ps, delivering 20-fold smaller energy compared to delivered at the first wavepacket arrival, still affecting the waiting-time dependence and the *T*_max_ value. The round trip for longer chains takes longer, making the amount of energy delivered after the wavepacket round trip even smaller. As a result, an increase in the apparent transport speed can be observed, even for a single band. However, the maximum apparent, *T*_max_-based speed is still limited by the maximum speed supported by the band.

The energy of the tag state with respect to the chain band, *E*_0_, affects minimally the *T*_max_(*R*) values for all chain lengths. Note that the DFT computations place the ν_ss,N3_ energy close to the top of the Tw band (Figure 1). However, the transport speed in short chains is affected by β_tag_, especially when the tag energy, *E*_0_, is close to that of the upper portion of the chain band. A small density of chain states in the short chains results in involvement of only a few chain states in the wavepacket. β_tag_ determines the number and identity of these states, affecting the transport speed: larger β_tag_ values make the apparent speed faster (reaching 18 Å/ps). Reducing β_tag_ below 5 cm^–1^ results in a lower apparent speed as well as larger *T*_max_ values, which significantly exceed the experimental values (∼4 ps already for *n* = 5). Small *k*_rep_ values (<1 ps^–1^) also lead to excessive *T*_max_ values, restricting *k*_rep_ to the range of 2–4 ps^–1^.

The parameters resulting in a good match with the experimental data at smaller *n* and reproducing the kink in the dependence, obtained using the guess and check method, are shown in the Figure 7 caption. The magenta line in Figure 7, obtained as the *T*_max_ of ∂*S*/∂*T*, represents the delay at which the energy flux into the reporter is maximal. The wavepacket speed determined from it, 26.4 Å/ps, represents the maximal speed supported by the band, which is ca. 26 Å/ps. Importantly, the dependence is essentially linear over the whole range of chain lengths, indicating that even if β_tag_ is smaller than the intrachain couplings (β and γ) multiple chain states participate in the wavepacket. While the modeling parameters obtained are not unique, the range of their acceptable values is not very wide.

For longer chains, the speed always gravitates to *V*_max_ staying within the 24–26 Å/ps range. No conditions were found in the modeling to obtain a faster speed than the maximal speed supported by the Tw band. Therefore, we conclude that the energy transport via the Tw band alone cannot describe the experiment for all chain lengths. We next consider the transport involving the Tw and W bands together and the transport involving the Tw, W, and Ro bands.

### 3.4 Transport Involving Two or Three Chain Bands

It was previously reported that the W modes of the chain are harmonically coupled to ν_ss_ with the interaction energy of ca. 10 cm^–1^.^36^ With the large width of the W band and because the ν_ss_ energy falls into the region of the highest slope of the W band, only a small mixing of ν_ss_ and W states occurs for short chains with *n* < 15, making the wavepacket on the W band insignificant for such chains. The Ro band is significantly broader than the W band, and ν_CN_ is located at the very steep portion of the Ro band (Figure 1) but their coupling is only ca. 9 cm^–1^, so the wavepacket within the Ro band is expected to contribute at even larger chain lengths.

**Figure 8:**
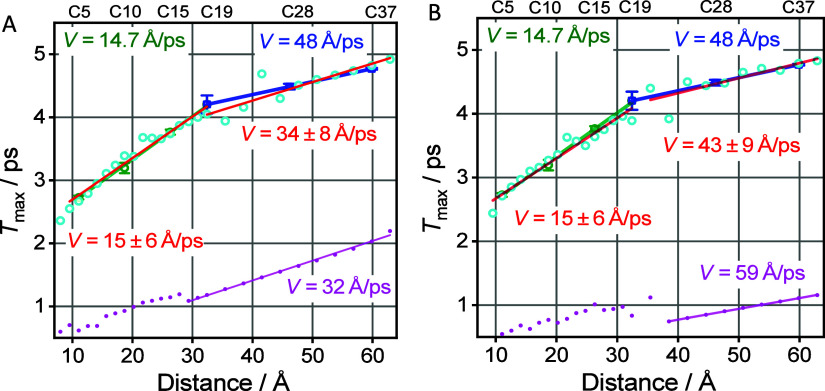
Tag-reporter distance dependence of *T*_max_ (cyan circles) computed for the transport via (A) two chain bands, Tw and W, and (B) three chain bands, Tw, W, and Ro. Linear fits of the modeling data for the *n* of 3–19 and *n* of 19–39 are shown with red lines and respective speeds are indicated as insets in red. The modeling parameters common for both panels were: *E*_0_ = (−25, 25, −40) cm^–1^, β_tag_ = (12, 10, 9) cm^–1^, *k*_deph_ = 0.4 ps^–1^, *k*_rlx_ = 0.6 ps^–1^, and *T*_c_ = 10 ps, where the first, second, and third values in parentheses are given for the Tw, W, and Ro bands, respectively. *k*_rep_ was at (3, 3) ps^–1^ for panel A and (4.5, 3, 4) ps^–1^ for panel B. The computed data (cyan circles) were shifted up by 0.6 ps (A) and 0.65 ps (B). The overall 2-band *T*-time kinetics were obtained by summing the kinetics for individual bands with fractions of 0.15 for Tw and 0.85 for W. The overall 3-band *T*-time kinetics were obtained by summing the kinetics for individual bands with the fractions of 0.063 for Tw, 0.104 for W, and 0.833 for Ro bands. Experimental data are also shown with green and blue symbols; the lines of the matching colors show the linear fits for short and long chains, taken from Figure 5.

The calculations were performed with two or three independent wavepackets and summing the energy delivered to the reporter site by each wavepacket. Each band was represented by its β and γ values (Table 1), interaction with the tag (β_tag_), dephasing (*k*_deph_) and relaxation (*k*_rlx_) rates, and the rate of populating the reporter site (*k*_rep_). In addition, contributions of each wavepacket, involving the relative efficiencies of the wavepacket formation, were used as fit parameters. Figure 8 shows the results of modeling by using two chain bands, Tw and W. The kink in the *T*_max_(*R*) dependence occurs when the transport via the W band becomes more efficient than that via the Tw band. As the maximum speed of the W band is smaller than the experimental at *n* > 19, the computed dependence does not fit the data well. Also, around the kink, the computed *T*_max_ values become somewhat “noisy” losing a monotonic increase with the chain length. The effect is easy to understand: when the density of W band states increases so that two W states can be found within ca. ± β_tag_ around the end-group state energy, the wavepacket efficiency increases, reducing the *T*_max_ value. However, at a slightly longer chain length, there will be only one W state within ± β_tag_ of the end-group state and the transport efficiency will drop resulting in a *T*_max_ value increase. Such fluctuations are clearly seen in the waiting-time dependence (Figure S31). The fluctuations will continue until about three W states are found in the vicinity of the end-group state, leading to a more stable efficiency of wavepacket formation in the W band. The *T*_max_ values obtained for ∂*S*/∂*T T*-dependence also experience a kink at *n* ∼ 15, which indicates the chain length at which the wavepacket in the W band starts to dominate over the wavepacket in the Tw band (Figure 8A, magenta dots). The group velocity determined for the *T*_max_ of ∂*S*/∂*T*, 32 Å/ps, corresponds approximately to the maximum speed supported by the W band (Table 1).

No conditions were found to fit the whole range of chain lengths using two bands, Tw and W. The transport speed for the longer chains was limited by the maximum speed of the W band, which is smaller than the experimental speed. Only by using all three bands, the data can be matched well (Figure 8B). The computed *T*_max_ points are noisy around the kink in the *T*_max_(*R*) dependence, indicating the chain length where the regime changes, as explained for the W band in Figure 8A. The magenta line starts at the chain length (∼40 Å) from where the transport via the Ro band dominates fully.

The parameters obtained for a good match are given in Figure 8 caption. The “noise” in the computed *T*_max_ values forced us to start the fit from *n* = 21. For *n* > 25 (>42 Å), the noise in *T*_max_ subsides and the speed gravitates toward the maximum speed of the Ro band.

Thus, the three-band model provides a reasonable explanation of the observed transport velocities. Yet, there are substantial fluctuations of transport times that exceed those observed experimentally. Potentially this problem can be resolved by including anharmonic interactions with low-frequency acoustic phonons, as outlined below as an alternative scenario of energy transport through optical bands.

### 3.5 Effect of Anharmonic Interactions with Acoustic Phonons

In the above consideration, we ignored the effect of anharmonic interaction. In particular, anharmonic interactions with gapless acoustic phonons, inevitably present in any chain and delocalized throughout it, can be involved in optical phonon transitions between different band states. Such transitions can include backward scattering accompanied by the overturn of the wavepacket propagation and replacing the transport mechanism from ballistic to diffusive. However, under the present condition of a sound velocity substantially exceeding the transport velocity, the scattering due to the interaction with longitudinal or torsional acoustic phonons is practically forbidden due to Cherenkov’s constraint.^46^ An interaction with transverse acoustic phonons is not subject to Cherenkov’s constraint because transverse phonons group velocity approaches zero at zero wavevectors.^47^ However, the bandwidth of the transverse acoustic phonons for alkyl chains, governed by interactions through covalent bonds, exceeds the bandwidth of the optical phonons. As a result, the strongest third-order anharmonic interaction can lead only to the forward scattering of optical phonons, while the backward scattering requires higher-order processes, which are orders of magnitude slower.^48^ If the initial group velocity of the optical phonon is smaller compared to its typical velocity within the band, then the fast-forward scattering redistributes this phonon between the band states with the same direction of propagation that will lead to the substantial increase of the phonon transport velocity with the time. This increase is equivalent to the increase of transport velocity for longer chains, as observed in the presented experiment. For example, if a wavepacket is created at the top of an optical chain band featuring a higher density of states but smaller “local” group velocity, anharmonic interaction with transverse acoustic phonons can cause the wavepacket to “migrate” to the middle of the band where the group velocity is much higher, still moving in the same direction. Most optical bands of the chain can be involved in such a process, which can result in a change in the observed transport speed.

Another opportunity for changing the transport speed is associated with a phonon transition from the given optical band to another with a lower energy. This transition can be due to anharmonic interactions involving three optical phonon bands or the solvent. Following this scenario, a wavepacket initiated in a twisting band can be transferred to the rocking band for longer chains. Of course, the propagation direction of the transferred phonon can vary; yet around half of them will keep propagating toward the reporter site and their further backscattering could be limited.^48^

## 4 Discussion

Azido groups offer multiple and potentially unique mechanisms of ballistic transport initiation in alkyl chains. Moreover, different mechanisms of initiation depend differently on the chain length. The transport mechanism via the Tw band involves two IVR steps; the ability to form a wavepacket in short chains (*n* < 16) is essentially independent of the chain length. The mechanism of the transport via the W and Ro bands is inefficient at short chains as the density of these chain states is insufficient to form a wavepacket. However, with an increase of the chain length, the wavepackets at W and Ro bands became increasingly more efficient, at the same time reducing the efficiency of wavepacket formation at the Tw band. This competitive switch occurs for the chain length of ca. 16–21; the switch results in a change of the apparent energy transport speed, which becomes ca. 3-fold faster.

Modeling of the transport involving only the Tw chain band showed that the kink in the *T*_max_(*R*) dependence can be reproduced under conditions in which wavepacket roundtrips contribute to the energy delivery to the reporter site. In this case, the kink occurs at chain lengths for which the roundtrip contributions become negligible. While the transport speed at small chain lengths and the kink location can be reproduced well via the modeling, the transport speed for longer chains cannot be reproduced. It is clearly shown that the apparent transport speed cannot exceed the maximal transport speed supported by the involved chain band. That is why incorporation of the W band, which starts to contribute at longer chains, cannot solve the problem either as the maximal transport speed supported by the W band is still smaller than the experimentally observed speed for longer chains. The Ro band supplies a significantly higher speed and adding a wavepacket on the Ro band enabled us to match the modeling with the experiment. We did not consider interference of different wavepackets and treated the three wavepackets as independent, as if they occur in different molecules. This approach is likely justified even if several wavepackets propagate in the same molecule because of orthogonality and different symmetry of different chain bands. Because the transport speeds supported by different chain bands differ significantly, the interference effects are expected to be small as the wavepackets arrive separately at the reporter site, especially for longer chains. Note that the time of the first arrival of each wavepacket is 2–3-fold smaller than the *T*_max_ values observed (compare the magenta line with the experimental data in Figures 7 and 8). That is because the observed maximum in the waiting time dependence is formed as a competition of the excess energy arriving at the end group and the end-group cooling.^36,45^

A “noise” in the *T*_max_ values was observed in the modeling at chain lengths for which a new band starts contributing to the energy transport but its density of states is still small. While we cannot say with certainty that such an effect is not observed in the experiment because of a small number of chain lengths measured, all experimental waiting time dependences measured did not show multiple peaks as in Figure S31. Possible reasons for smoothing in the experimental data is in the diversity of the molecular structures of the chain, which includes deviations of the angles and bond lengths in the chain from those corresponding to the potential energy minima for isolated chains.

## 5 Conclusions

It is shown that the transport speed of optical phonons propagating through alkyl chains is getting faster with increasing chain length. The observed changes in the apparent transport rate for longer chains, observed experimentally, suggested a switch of the transport regime. Modeling involving the numerical solution of the Liouville equation for the density matrix revealed that involvement of a single chain band (Tw) or two bands (Tw and W) cannot describe the experiment. The obtained modeling parameters, *k*_deph_ = 0.4 ps^–1^ and *k*_rlx_ = 0.6 ps^–1^ revealed that multiple roundtrips involving wavepacket reflections at the chain ends play a role only to short distances. While the wavepacket is expected to undergo some broadening and deformation due to nonlinearity of the dispersion curves for optical bands, the modeling suggests that the wavepacket can potentially exist in the chain for a long time, exceeding 10 ps, given that the dephasing and relaxation of the chain states are slow. These observations open an opportunity for sending the wavepacket to much larger distances at lower temperatures. The study provides critical testing of the mechanisms of ballistic transport in oligomeric chains. Additional mechanisms of the transport initiation appearing for longer chains are more efficient, as they eliminate IVR steps in making the wavepackets in W and Ro bands. The increased speed causes a decrease in losses in the chain, thus also increasing the overall transport efficiency.

## Supporting Information Available

additional figures and details of 2DIR measurements, synthesis and characterization of the test compounds, and vibrational dynamics modeling (PDF)
