# Supplementary material for: Ballistic Energy Transport via Long Alkyl Chains: A New Initiation Mechanism
Source: J Phys Chem B. 2024 Sep 2;128(36):8788–96. doi: 10.1021/acs.jpcb.4c03386 (PMC11403685; doi:10.1021/acs.jpcb.4c03386)
Supplement: Supplementary file 1 — jp4c03386_si_001.pdf [file jp4c03386_si_001.pdf]

Supporting Information

Ballistic Energy Transport via Long Alkyl Chains: A New Initiation Mechanism

Sithara U. Nawagamuwage, Elliot S. Williams, Md Muhaiminul Islam, Igor V. Parshin, Alexander L. Burin, Nathalie Busschaert, Igor V. Rubtsov*

Department of Chemistry, Tulane University, New Orleans, Louisiana 70118, United States

irubtsov@tulane.edu

S1. Synthesis

S1.1. Reaction Schemes

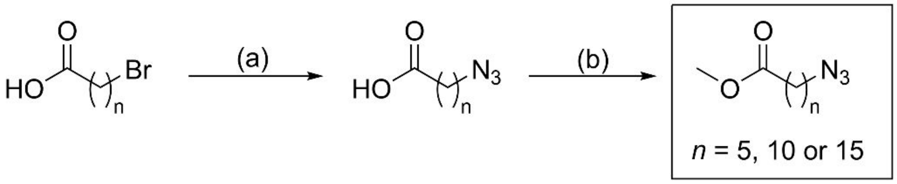

Scheme S1. Short azide fatty esters reaction scheme. The shortest chain was sourced as ω-azidofatty acid chain. Az5ME (n = 5, 99% yield), az10ME (n = 10, 77% yield), and az15ME (n = 15, 86% yield). (a) NaN_3 (4 eq.), DMF, 20 h, r.t. (b) dilute MeOH/HCl, toluene/MeOH, 50 °C, 18 h.

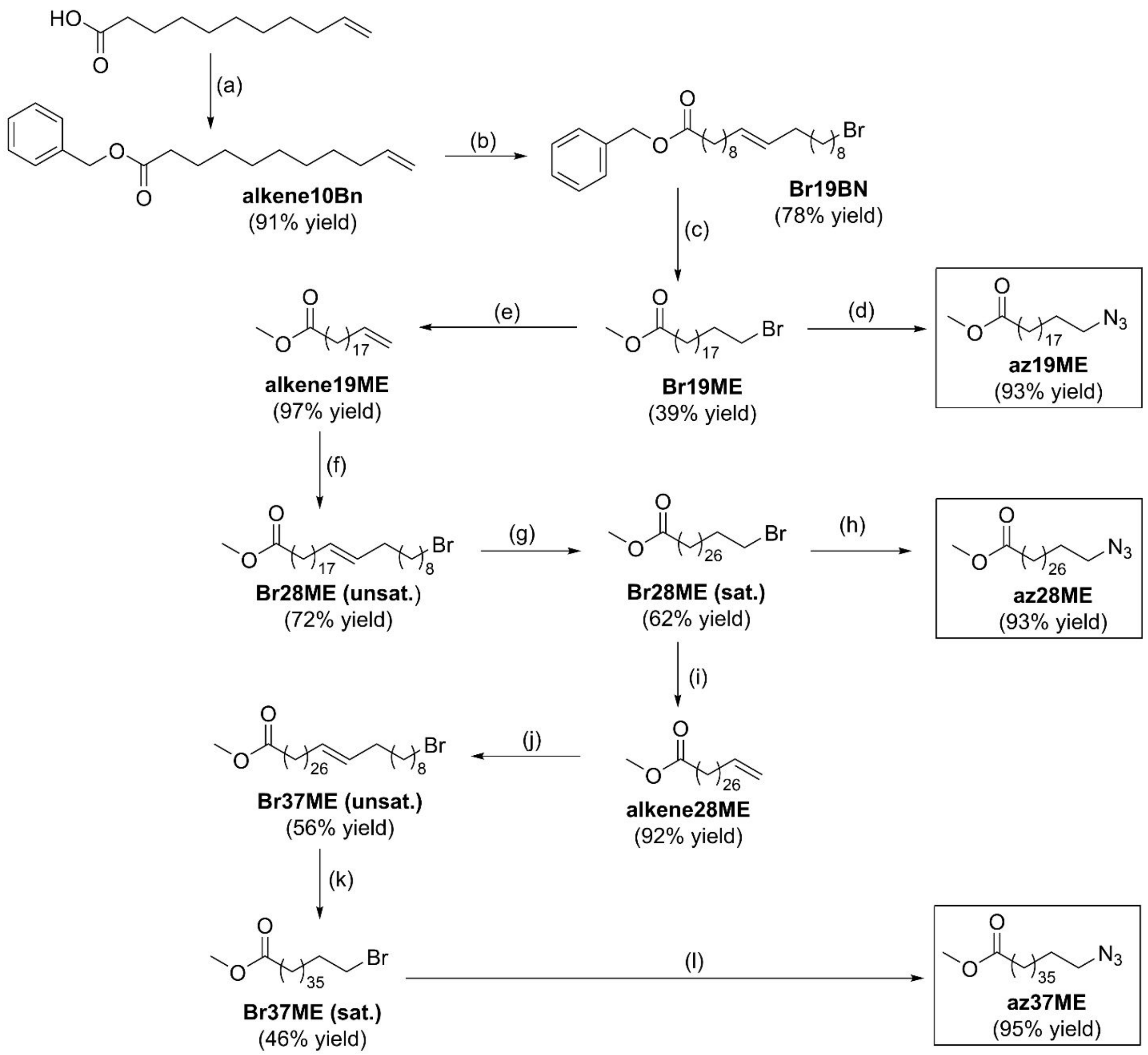

Scheme S2. Long azide fatty esters reaction scheme: (a) 1. NaOH, MeOH, 30 min, r.t., 2. BnBr (1.2 eq), DMF, 90 ˚C, 18 h. (b) 11-Bromoundecene (10 eq.), 40 ˚C, 20 h. (c) H_2, 1:2 MeOH/ethyl acetate, Pd/C cat. (10 wt. %). (d) 4 eq. NaN_3, DMF, r.t. 20 h (e) $Pd_2(dba)_3$, $[HP(t\text{-}Bu)_2Me)]BF_4$, dioxane, *t*-BuOK, Cy_2NH. (f) 11-Bromoundecene (10 eq.), DCM, 40 ˚C, 20 h. (g) H_2, 1:2 MeOH/ethyl acetate, Pd/C cat. (10 wt. %) (h) 4 eq. NaN_3, DMF, r.t., 20 h. (i) $Pd_2(dba)_3$, $[HP(t\text{-}Bu)_2Me)]BF_4$, dioxane, *t*-BuOK, Cy_2NH. (j) 11-Bromoundecene (20 eq.) DCM, 40 ˚C, 20 h. (k) H_2, 1:2 MeOH/ethyl acetate, Pd/C cat. (10 wt. %). (l) 72 eq. NaN_3, DMF, r.t. 72 h.

S1.2. Synthesis Procedures

S1.3. Compound names are those generated by ChemDraw 16.0.1.4 following IUPAC nomenclature. Solvents, reagents, and inorganic salt were supplied from AA Blocks, Abachemscene, Sigma-Aldrich, TCI America, and Combi-Blocks. Reagents were used without further purification unless specified. Inert reactions were conducted either under an ultra-high purity argon or ultra-high purity nitrogen atmosphere in oven-dried glassware. Flash column chromatography was carried out using SiliaFlash P60 (40-63 µm, 230-400 mesh) or basic alumina. Thin-layer chromatography was carried out using silica gel TLC plates with fluorescent indicators, visualized under UV light (254 nm), or stained with iodine, ninhydrin, or permanganate solutions. ^{1}H NMR and ^{13}C NMR, spectra were collected on either a Bruker 500 MHz NMR, Bruker Avance Neo 400 MHz NMR, Varian Unity Inova 400 MHz NMR, or a Bruker Avance 300 MHz NMR spectrometer. ^{13}C NMR spectra were collected proton decoupled and visualized in Mestrenova. Chemical shifts (δ) are reported in parts per million (ppm) and calibrated to the residual solvent peak in $CDCl_3$ δ = 7.26 (^{1}H NMR) and 77.2 ppm (^{13}C NMR) or DMSO-d_6 δ = 2.50 (^{1}H NMR) and 39.5 ppm (^{13}C NMR). Coupling constants (*J*) are reported in Hertz (Hz) and rounded to the nearest 0.5 Hz. The following abbreviations are used for spin multiplicity: s = singlet, d = doublet, t = triplet, q = quartet, p = pentet, ddt = doublet of doublet of triplets, m = multiplet, and br. = broad. Infrared (IR) spectra were recorded on a Nexus 670 Avatar FTIR spectrometer; only selected maximum peaks (ν_{max}, cm^{-1}) are reported. Electron spray ionization (ESI) mass spectra were recorded on a Bruker MicroTOF.

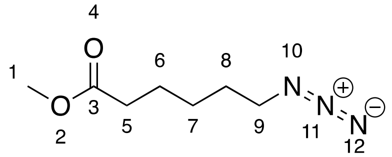

Methyl 6-azidohexanoate,ν (az5ME). 6-Azidohexanoic acid (298 mg, 1.896 mmol) was dissolved in 2 mL toluene, 20 mL methanol, and 3 mL of dilute MeOH/HCl solution (made by adding 10 mL concentrated HCl to 40 mL of MeOH). The solution was heated to 50 °C and stirred overnight for 20 h. The reaction was sealed with a glass stopper to prevent the solvent from evaporating. After 20 h, the reaction was cooled to room temperature, and the solvent volume was reduced using the roto-evaporator leaving the crude and some toluene. The crude was dissolved in 20 mL hexanes and transferred to a 150 mL extraction funnel. 20 mL of water was also added to extraction funnel. The product was extracted into the hexanes layer and collected in a beaker. The extraction was repeated twice more with 20 mL of hexanes. The collected organic layer was dried over $MgSO_4$ and filtered to remove the drying agent. The dried hexanes solution was roto-evaporated to remove the solvent, leaving behind a clear oil. The oil was further evaporated with a gentle stream of argon gas for 30 minutes. The product is a volatile clear oil that should not be placed under high vacuum. (320 mg, 1.87 mmol, 99% yield). The spectra were in agreement with literature reported data.[1] ^{1}H NMR (400 MHz, $CDCl_3$, ppm) δ 3.66 (3H, s, **1**), 3.26 (2H, t, *J*=7.0 Hz, **9**), 2.32 (2H, t, *J*=7.5 Hz, **5**), 1.70 – 1.55 (2H, m, **6, 8**), 1.47 – 1.31 (2H, m, **7**). ^{13}C NMR (101 MHz, $CDCl_3$, ppm) δ 174.0 (**3**), 51.6 (**1**), 51.3 (**9**), 33.9 (**5**), 28.7 (**7**), 26.3 (**8**), 24.5 (**6**). IR (neat): ν (cm^{-1}) = 2946, 2865, 2091, 1735, 1346, 1352, 1251, 1001, 1173, 1154, 1098, 1009.

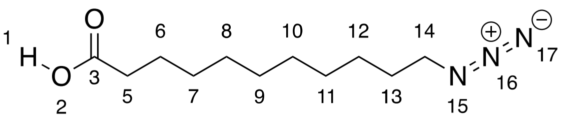

11-Azidoundecanoic acid, (az10OH). 11-Bromoundecanoic acid (1.361g, 5.132 mmol, 1 eq) and NaN_3 (1.465 g, 22.538 mmol, 4.4 eq.) were dissolved in 25 mL DMF in a 100 mL round bottom flask equipped with a magnetic stir bar. The reaction was stirred at r.t. for 20 h under ambient atmosphere. Afterwards, the solution was transferred into a 250 mL extraction funnel. 50 mL ethyl acetate was added to the round bottom flask and then transferred into the extraction funnel ensuring efficient transfer. 100 mL H_2O was added to the extraction funnel. The solution was then acidified with 5% HCl v/v solution. The aqueous layer was collected, and the organic layer was washed two more times with 100 mL dilute HCl solution. The organic layer was collected into a 100 mL round bottom flask, the solvent was removed via roto-evaporation, and then further dried under high vacuum. The product was obtained as a clear oil that required no further purification. (1.116 g, 4.910 mmol, 96% yield). ^{1}H NMR (300 MHz, $CDCl_3$, ppm) δ 3.25 (2H, t, *J*=7.0 Hz, **16**), 2.35 (2H, t, *J*=7.5 Hz, **5**), 1.81 – 1.47 (4H, m, **6, 15**), 1.45 – 1.11 (12H, m, **7, 8, 9, 10, 11, 12, 13, 14**) The spectral features were in agreement with literature reported values.[2]

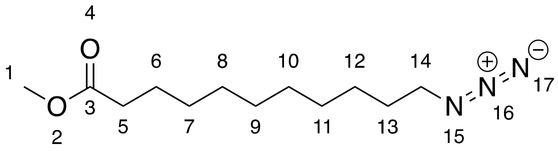

Methyl 11-azidoundecanoate, (az10ME). 11-azidoundecanoic acid (**az10OH**) (1.116 g, 4.901 mmol) was dissolved in 2 mL toluene, 20 mL methanol, and 3 mL of dilute MeOH/HCl solution (made by adding 10 mL concentrated HCl to 40 mL of MeOH). The solution was heated to 50 °C and stirred overnight for 18 h. The reaction was not sealed so the solvent mostly evaporated. 15 mL MeOH was added to increase the volume and then transferred into a 150 mL extraction funnel. The product was extracted 2x with 25 mL hexanes. The hexane layer was transferred to a round bottom flask, evaporated via roto-evaporation and further dried under high vacuum. The product was a pure clear oil that required no further purification (946 mg, 3.91 mmol, 80% yield). The spectra were in agreement with literature reported data.[2] ^{1}H NMR (400 MHz, $CDCl_3$, ppm) δ 3.75 – 3.55 (3H, s, **1**), 3.24 (2H, t, *J*=7.0 Hz, **14**), 2.29 (2H, t, *J*=7.5 Hz, **5**), 1.68 – 1.50 (4H, m, **6, 13**), 1.27 (12H, s, **7, 8, 9, 10, 11, 12**). ^{13}C NMR (101MHz, $CDCl_3$, ppm) δ 174.4 (**2**), 51.6 (**14**), 51.5 (**1**), 34.2 (**5**), 29.7 – 28.8 (m, **7, 8, 9, 10, 11, 12**), 26.8 (**13**), 25.0 (**6**) IR (neat): ν (cm^{-1}) = 2926, 2855, 2092, 1738, 1457, 1436, 1251, 1196, 1170, 1103.

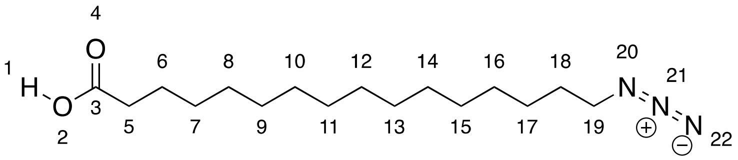

16-Azidohexadecanoic acid, (az15OH). 16-Bromohexadecanoic acid (465 mg, 1.386 mmol, 1 eq) and NaN_3 (360 mg, 5.538 mmol, 4 eq) were dissolved in 25 mL DMF in a 100 mL round bottom flask with a stir bar. The reaction was stirred at room temperature for 20 h. After 20 h the reaction was worked up by dissolving the product in ethyl acetate and washing with water to remove DMF and NaN_3. The reaction was incomplete according to NMR, so the crude was redissolved in 25 mL of DMF and an additional 4 eq. NaN_3 was added to the flask. The reaction was stirred for an additional 20 h at room temperature. Afterwards, the solution was transferred into a 250 mL extraction funnel. 50 mL ethyl acetate was added to the round bottom and then transferred into the extraction funnel. 100 mL H_2O was added to the extraction funnel. The solution was then acidified with 5% HCl v/v solution. The aqueous layer was drained and repeated two more times with 100 mL dilute HCl solution. The organic layer was collected into a 100 mL round bottom flask and the solvent was removed via roto-evaporation, and then further dried under high vacuum. The product was obtained as a tan solid that required no further purification. NMR spectra were in agreement with those previously reported in literature.[3] (392 mg, 1.32 mmol, 96% yield) ^{1}H NMR (300 MHz, $CDCl_3$,

ppm) δ 3.2 (2H, t, *J*=7.0 Hz, **5**), 2.3 (2H, t, *J*=7.5 Hz, **19**), 1.6 (4H, m, **6, 18**), 1.3 (22H, s, **7, 8, 9, 10, 11, 12, 13, 14, 15, 16, 17**). ^{13}C NMR (75 MHz, $CDCl_3$, ppm) δ 180.4 (**3**), 51.6 (**19**), 34.2 (**5**), 29.9 – 28.7 (m, **7, 8, 9, 10, 11, 12, 13, 14, 15, 16, 17**), 26.9 (**18**), 24.8 (**6**)

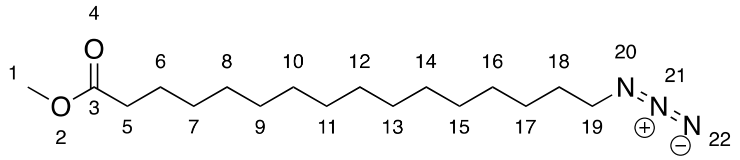

Methyl 16-azidohexadecanoate, (az15ME). 16-Azidohexadecanoic acid (**az15OH**) (392 mg, 1.32 mmol) was dissolved in 2 mL toluene, 20 mL methanol, and 3 mL of dilute MeOH/HCl solution (made by adding 10 mL concentrated HCl to 40 mL of MeOH). The solution was heated to 50 °C and stirred overnight for 18 h. The reaction was sealed with a glass stopper to prevent the solvent from evaporating. The solution was cooled to room temperature and transferred into a 150 mL extraction funnel. The product was extracted with 50 mL hexanes split between two additions. The hexane layers were evaporated via roto-evaporation, and further dried under high vacuum. The product obtained was a pure white solid that required no further purification (373 mg, 1.20 mmol, 90% yield) ^{1}H NMR (300 MHz, $CDCl_3$, ppm) δ 3.66 (s, 3H, **1**), 3.25 (t, *J* = 7.0 Hz, 2H, **19**), 2.30 (t, *J* = 7.5 Hz, 2H, **5**), 1.73 – 1.56 (m, 4H, **6**, **18**), 1.44 – 1.13 (m, 22H, **7**, **8**, **9**, **10**, **11**, **12**, **13**, **14**, **15**, **16**, **17**). ^{13}C NMR (101 MHz, $CDCl_3$): 174.5 (**3**), 51.6 (**1**), 51.6 (**19**), 34.3 (**5**), 29.9 – 28.9 (m, **7, 8, 9, 10, 11, 12, 13, 14, 15, 16, 17**), 26.9 (**18**), 25.1 (**6**).). IR (neat): ν (cm^{-1}) = 2923, 2853, 2093, 1739, 1457, 1436, 1351, 1251, 1197, 1169, 1111, 1015.

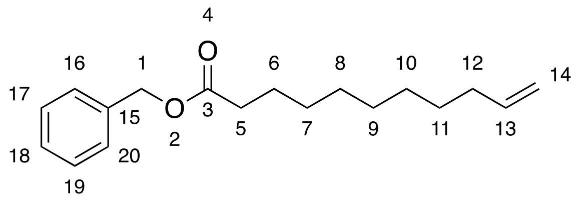

Benzyl undec-10-enoate, (alkene10BN). NaOH (5.13 g, 0.128 mol, 1.0 eq.) was dissolved in 70 mL MeOH. Once fully dissolved, undec-10-enoic acid (23.62 g, 0.128 mol, 1.0 eq) was slowly added to the methanol solution while stirring. A precipitate had formed after 40 mins of stirring. MeOH was removed via roto-evaporation and the flask was dried on the high vacuum for one hour. Benzyl bromide (23.229 g, 0.135 mol, 1.1 eq) was dissolved in 200 mL DMF. This solution was transferred into the sodium salt of the fatty acid and the solution was heated and stirred at 90 °C for 18 h. After 18 h, the DMF solution was transferred to a 500 mL extraction funnel, 100 mL ethyl acetate was added and washed with 100 mL DI H_2O (three times). The organic layer was transferred into a round bottom flask and the solvent was removed via roto-evaporation and dried on high vacuum. The crude product was then purified with a silica column using hexanes as mobile phase. The purified product was dried on high vacuum. In the case of excess benzyl bromide: the product was treated with triethylamine (0.4 eq) for 24 h with stirring. The resultant TEA-Br salt was filtered off leaving a pure clear liquid product. (32.15 g, 0.117 mols, 91% yield) ^{1}H NMR (300 MHz, $CDCl_3$, ppm) δ 7.46 – 7.30 (5H, m, **16,17,18,19,20**), 5.83 (1H, ddt, *J*=17.0, 10.0, 6.5 Hz, **13**), 5.14 (2H, s, **1**), 5.08 – 4.86 (2H, m, **14**), 2.38 (2H, t, *J*=7.5 Hz, **5**), 2.08 (2H, q, *J*=7.0 Hz, **12**), 1.77 – 1.55 (2H, m, **6**), 1.42 – 1.23 (10H, m, **7,8,9,10,11**). ^{13}C NMR (101 MHz, $CDCl_3$, ppm) δ 173.6 (**3**), 139.2 (**13**), 136.2 (**15**), 128.6 (**17, 19**), 128.3 – 128.1 (m, **16, 18, 20**), 114.2 (**14**), 66.1 (**1**), 34.4 (**5**), 33.8 (**12**), 29.5 – 28.7 (m, **7, 8, 9, 10, 11**), 25.0 (**6**). IR (neat): ν (cm^{-1}) = 3065, 3032, 2926, 2854, 1735, 1640, 1455, 1158, 993, 908. HRMS (ESI+) for $C_{18}H_{26}O_2$ $[M+H]^+$: *m/z* = 275.1988 (observed), 275.2011 (calculated).

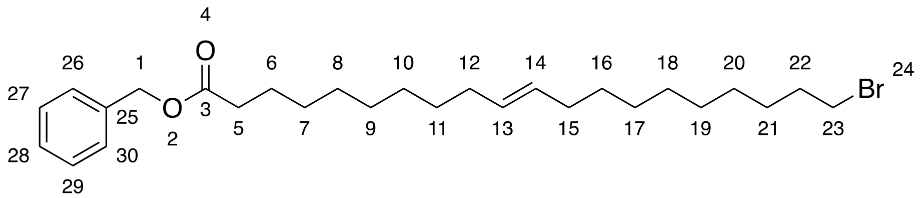

Benzyl (*E*)-20-bromoicos-10-enoate, (Br19BN). Benzyl undec-10-enoate (**alkene10BN**) (25.1 g, 91.5 mmol, 1.0 eq.) was added to 11-bromoundec-1-ene (213.0 g, 913 mmol, 10.0 eq) into a 500 mL round bottom flask. This reaction was performed neat, without the use of solvents. The flask was evacuated, backfilled with argon gas, and subjected to 3 freeze-thaw cycles. While freezing, the flask was evacuated. While thawing, the flask was backfilled with argon gas. Once thawed, 2.0 g of Grubbs second generation catalyst was added and a reflux condenser with a vacuum stopcock adaptor was attached to the round bottom flask. The flask was evacuated again, backfilled with argon gas, and subjected to one more freeze thaw cycle. Once thawed, the round bottom flask was heated to 111 °C and refluxed overnight. After 20 h, the round bottom flask was removed from heat and allowed to cool. The crude oil was loaded neat onto a large silica column with a hexane mobile phase. The 11-bromoundec-1-ene starting material was eluted with hexanes. The metathesis product eluted with 10% ethyl acetate in hexanes. The pure fractions were collected in a round bottom flask and the solvent was evaporated. The product was obtained as an oil (34.0 g, 70.9 mmol 78% yield). ^{1}H NMR (300 MHz, $CDCl_3$, ppm) δ 7.4 – 7.3 (5H, m, **26, 27, 28, 29, 30**), 5.5 – 5.3 (2H, m, **13, 14**), 5.1 (2H, s, **1**), 3.4 (2H, t, *J*=7.0 Hz, **23**), 2.3 (2H, t, *J*=7.5 Hz, **5**), 2.1 – 1.9 (4H, m, **12, 15**), 1.8 (2H, p, *J*=7.0 Hz, **22**), 1.6 (2H, t, *J*=7.0 Hz, **6**), 1.5 – 1.4 (2H, m, **21**), 1.4 – 1.1 (20H, m, **7, 8, 9, 10, 11, 16, 17, 18, 19, 20**). ^{13}C NMR (101 MHz, $CDCl_3$, ppm) δ 173.8 (**3**), 136.2 (**25**), 130.4 (t, *J*=5.0 Hz, **13, 14**), 128.6 (**26, 30**), 128.3 (**27, 28, 29**), 66.2 (**1**), 34.5 (**5**), 34.2 (**23**), 33.0 (**22**), 32.7 (**12, 15**), 30.0 – 28.5 (m, **7, 8, 9, 10, 11, 16, 17, 18, 19, 20**), 28.3 (**21**), 25.1 (**6**). IR (neat): *ν (*cm^{-1}) = 3065, 3031, 2924, 2853, 1737, 1456, 1436, 1353, 1165, 967. HRMS (ESI+) for $C_{27}H_{43}BrO_2$ [M+H]$^+$: *m/z* = 479.2519 (calculated), 479.2527 (observed).

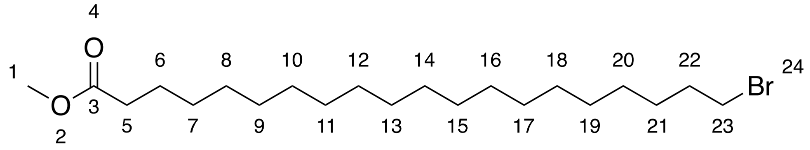

Methyl 20-bromoicosanoate, (Br19ME). Benzyl 20-bromoicosanoate (**Br19BN)** (3.124 g, 6.51 mmol, 1.0 eq.) was dissolved in 30 mL 1:2 ethyl acetate:methanol and Pd/C (10 wt. %) (0.161 g, 1.51 mmol, 0.2 eq) was added to the 100 mL round bottom flask. The flask was evacuated and backfilled with argon gas (3 cycles). The flask was evacuated, and a soccer ball inflated with hydrogen gas was attached. The reaction stirred at room temperature for 20 h. After 20 h, the reaction solution was gravity filtered through a celite plug to remove Pd/C catalyst. The celite plug was washed with 50 mL hexanes to ensure the product was removed. The filtrate was evaporated leaving a crude oil, which was dissolved in methanol by heating the solution to 50 °C. Once dissolved, the solution was cooled to room temperature forming a white precipitate, and then further cooled to 8 ˚C. The precipitate was filtered off and collected with chloroform. The chloroform was evaporated off and the solid was dried under high vacuum at room temperature overnight (1.024 g, 2.53 mmol, 39 % yield). ^{1}H NMR (400 MHz, $CDCl_3$, ppm) δ 3.66 (3H, s, **1**), 3.40 (2H, t, *J*=7.0 Hz, **23**), 2.30 (2H, t, *J*=7.5 Hz, **5**), 1.85 (2H, p, *J*=7.0 Hz, **22**), 1.68 – 1.57 (2H, m, **6**), 1.42 (2H, p, *J*=7.0 Hz, **21**), 1.30 – 1.21 (28H, m, **7, 8, 9, 10, 11, 12, 13, 14, 15, 16, 17, 18, 19, 20**) ^{13}C NMR (101 MHz, $CDCl_3$, ppm) δ.5 (**3**), 51.6 (**1**), 34.3 (**5**), 34.2 (**23**), 33.0 (**22**), 29.9 – 29.1 (m, **8, 9, 10, 11, 12, 13, 14, 15, 16, 17, 18, 19, 20**), 28.9 (**7**), 28.3 (**21**), 25.1 (**6**). IR (neat): *ν (*cm^{-1}) = 2915, 2847, 1734, 1473, 1435, 1379, 1200, 1170, 1117, 980, 884. HRMS (ESI+) for $C_{21}H_{41}BrO_2$ [M+H]$^+$: *m/z* = 407.2350 (calculated) 407.2336 (observed).

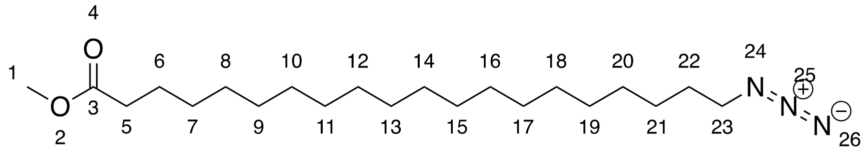

Methyl 20-azidoicosanoate, (az19ME). Br19ME (214 mg, 0.528 mmol, 1.0 eq), NaN_3 (190 mg, 2.92 mmol, 5.5 eq.) and 20 mL DMF were added to a 50 mL round bottom flask with a stir bar. The reaction was stirred at room temperature for 20 h. The reaction was then transferred to a 250 mL separatory funnel. 50 mL ethyl acetate was added and washed 3x with 100 mL DI H_2O to remove DMF. The organic layer was transferred to a round bottom flask and the solvent was evaporated, leaving behind a clear oil. The oil was further evaporated under a gentle stream of argon gas leaving behind **az19ME** as a white solid, which was dried overnight under high vacuum (180 mg, 0.490 mmol, 93% yield). ^1H NMR (300 MHz, $CDCl_3$, ppm) δ 3.65 (3H, s, **1**), 3.24 (1H, t, *J*=7.0 Hz, **23**), 2.29 (2H, t, *J*=7.5 Hz, **5**), 1.69 – 1.51 (4H, m, **6, 22**), 1.24 (30H, s, **7, 8, 9, 10, 11, 12, 13, 14, 15, 16, 17, 18, 19, 20, 21**) ^{13}C NMR (101 MHz, $CDCl_3$, ppm) δ 174.5 (**3**), 51.7 (**23**), 51.6 (**1**), 34.3 (**5**), 30.0 – 29.4 (m, **8, 9, 10, 11, 12, 13, 14, 15, 16, 17, 18, 19, 20, 21**), 29.3 (**7**), 26.9 (**22**), 25.1 (**6**). IR (neat): ν (cm^{-1}) = 2915, 2848, 2096, 1737, 1473, 1262, 1378, 1170, 884. HRMS (ESI+) for $C_{21}H_{41}N_3O_2$ $[M+Na]^+$: *m/z* = 390.3096 (calculated), 390.3091 (observed).

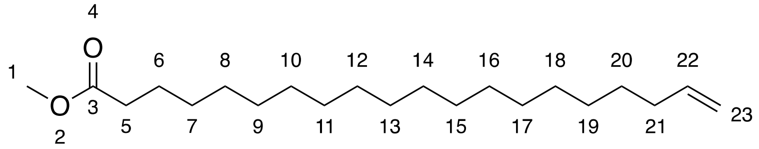

Methyl icos-19-enoate, (alkene19ME). $Pd_2(dba)_3$ (214 mg, 234 μmol, 4 mol%) $[HP(t\text{- }Bu)_2Me)]BF_4$ (233 mg, 803 μmol, 14 mol%), and a stir bar were added to an oven-dried 50-mL 3-neck flask. The flask was sealed with two rubber septa and a stopcock vacuum adaptor. The flask was then evacuated and backfilled with argon (3 cycles). The flask was detached from the argon line, and 10 mL dioxane (anhydrous), *t*-BuOK (1.5 mL of a 1 M solution in THF, 1.5 mmol, 0.25 eq.) and Cy_2NH (1.4 mL, 7.2 mmol, 1.2 eq.) were added sequentially via syringe. The puncture holes of the septa were sealed with vacuum grease, and the mixture was stirred at r.t. for 2 h. **Br19ME** (2.438 g, 6.01 mmol, 1.0 eq) was placed in a vial with a septum lid. The vial was purged with argon for 30 min and dissolved in 2 mL anhydrous dioxane. After 2 h of reacting the pre-catalyst, **Br19ME** was transferred into the reaction via a long needle syringe. An additional 2 mL anhydrous dioxane was used to wash the vial and ensure an efficient transfer. The reaction mixture was stirred for 24 h at room temperature. The reaction solution was then vacuumed filtered through a silica plug followed by an additional 100 mL of 5% Et_2O in hexanes. The filtrate was evaporated and dried on high vacuum. The product was considered pure enough to do the next reaction (1.897 g, 5.86 mmol, 97% yield). ^1H NMR (400 MHz, $CDCl_3$, ppm) δ 5.81 (1H, ddt, *J*=17.0, 10.0, 7.0 Hz, **22**), 5.10 – 4.78 (2H, m, **23**), 3.66 (3H, s, **1**), 2.29 (2H, t, *J*=7.5 Hz, **5**), 2.03 (2H, q, *J*=7.0 Hz, **21**), 1.65 – 1.59 (2H, m, **6**), 1.41 – 1.17 (28H, m, **7, 8, 9, 10, 11, 12, 13, 14, 15, 16, 17, 18, 19, 20**). ^{13}C NMR (101 MHz, $CDCl_3$, ppm) δ 174.4 (**3**), 139.4 (**22**), 114.2 (**23**), 51.6 (**1**), 34.3 (**5**), 34.0 (**21**), 30.2 – 28.4 (m, **7, 8, 9, 10, 11, 12, 13, 14, 15, 16, 17, 18, 19, 20**), 25.1 (**6**). IR (neat): ν (cm^{-1}) = 3065, 2915, 2848, 1739, 1641, 1466, 1435, 1365, 1166, 992, 909. HRMS (ESI+) for $C_{21}H_{40}O_2$ $[M+H]^+$: *m/z* = 325.3107 (calculated), 325.3079 (observed).

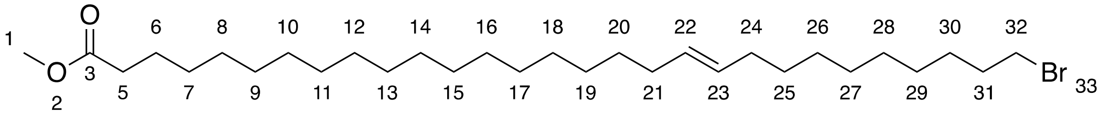

Methyl (*E*)-29-bromononacos-19-enoate, (Br28ME (unsat.)). Methyl icos-19-enoate (**alkene19ME**) (1.897 g, 5.855 mmol, 1.0 eq), 11-bromoundec-1-ene (14.26 g, 61.15 mmol, 10.44 eq), and Grubbs Second

Gen. Cat. (244 mg, 0.287 mmol, 5 mol%) were added into a 100 mL round bottom flask with a stir bar and dissolved in 40 mL DCM. A reflux condenser was attached to the flask and a vacuum adaptor stopcock was attached to the condenser. The reaction vessel was placed under high vacuum and evacuated, backfilled with argon gas, and subjected to 3 freeze-thaw cycles. While freezing, the flask was evacuated. While thawing, the flask was backfilled with argon gas. Once thawed, the round bottom flask was heated to 40 °C and refluxed overnight. After 20 h, the round bottom flask was removed from heat and allowed to cool. The crude oil was purified via column chromatography with a silica stationary phase and a gradient hexanes:ethyl acetate mobile phase. The 11-bromoundec-1-ene starting material eluted with 100% hexanes. The metathesis product eluted with 10% ethyl acetate in hexanes (2.220 g, 4.202 mmol, 72% yield). ^{1}H NMR (400 MHz, $CDCl_3$, ppm) δ 5.5 – 5.3 (2H, m, **22**, **23**), 3.7 (3H, s, **1**), 3.4 (2H, t, *J*=7.0 Hz, **32**), 2.3 (2H, t, *J*=7.5 Hz, **5**), 2.1 – 1.9 (4H, m, **21, 24**), 1.9 (2H, p, *J*=7.0 Hz, **31**), 1.7 – 1.6 (2H, m, **6**), 1.5 – 1.4 (2H, m, **30**), 1.4 – 1.2 (38H, m, **7, 8, 9, 10, 11, 12, 13, 14, 15, 16, 17, 18, 19, 20, 25, 26, 27, 28, 29**) ^{13}C NMR (101 MHz, $CDCl_3$, ppm) δ 174.5 (**3**), 130.9 – 130.2 (m, **22, 23**), 51.6 (**1**), 34.3 (**32**), 34.2 (**5**), 33.0 (**31**), 32.8 (**21, 24**), 30.0 – 28.8 (m, **7, 8, 9, 10, 11, 12, 13, 14, 15, 16, 17, 18, 20, 25, 26, 27, 28, 29**), 28.3 (**30**), 25.1 (**6**). IR (neat): *ν* (cm^{-1}) = 3143, 2917, ,2849, 1739, 1646, 1463, 1435, 1377, 1168, 963. HRMS (ESI+) for $C_{30}H_{57}BrO_2$ [M+Na]$^+$: *m/z* = 553.3424 (calculated), 553.3385 (observed).

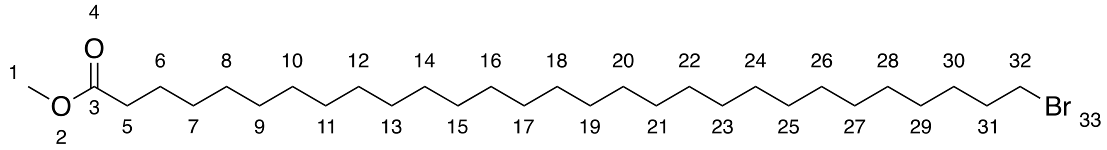

Methyl 29-bromononacosanoate, **(Br28ME (sat.)).** Methyl (*E*)-29-bromononacos-19-enoate (**Br28ME (unsat.)**) (645 mg, 1.22 mmol) was dissolved in 30 mL 1:1 ethyl acetate:methanol in a 100 mL round bottom flask with a magnetic stirrer. A small scoop of Pd/C (10 wt. % loading) was added to the reaction. The reaction was sealed with a vacuum adaptor equipped with a stopcock. The reaction flask was evacuated and backfilled with argon gas three times. The flask was evacuated once more and attached to a soccer ball pressurized with hydrogen gas. The reaction was stirred overnight at r.t. and monitored by TLC to ensure completion. The TLC was developed with 2% ethyl acetate in hexanes and visualized with potassium permanganate. Once complete, the solution was gravity filtered through a celite plug followed by 100 mL hexanes to collect the product. The filtrate was transferred to a round bottom flask and evaporated leaving a waxy crude. The crude was dissolved in ethyl acetate and precipitated using methanol. Once the solid precipitated, the solution was heated to 50 °C using a water bath to redissolve the solid. Once dissolved, a precipitate slowly crashed out of solution at r.t., which was removed from solution via vacuum filtration using a fine glass frit Buchner funnel. The solid was collected off the glass frit by dissolving the solid in chloroform. The chloroform solution was transferred into a round bottom flask and the solvent was removed via roto evaporation. A clear oil was obtained and further evaporated under a gentle stream of argon gas leaving behind **Br28ME (sat.)** as a pure white powder (400 mg, 0.752 mmol, 62% yield). ^{1}H NMR (400 MHz, $CDCl_3$, ppm) δ 3.66 (3H, s, **1**), 3.40 (2H, t, *J*=7.0 Hz, **32**), 2.29 (2H, t, *J*=7.5 Hz, **5**), 1.85 (2H, p, *J*=7.0 Hz, **31**), 1.61 (2H, p, *J*=7.5 Hz, **6**), 1.49 – 1.36 (2H, m, **30**), 1.26 (46H, s, **7, 8, 9, 10, 11, 12, 13, 14, 15, 16, 17, 18, 19, 20, 21, 22, 23, 24, 25, 26, 27, 28, 29**). ^{13}C NMR (101 MHz, $CDCl_3$, ppm) δ 174.4 (**3**), 51.6 , (**1**), 34.3 (**5**), 34.1 (**32**), 33.0 (**31**), 29.9 – 29.2 (m, **8, 9, 10, 11, 12, 13, 14, 15, 16, 17, 18, 19, 20, 21, 22, 23, 24, 25, 26, 27, 28, 29**), 28.9 (**7**), 28.3 (**30**), 25.1 (**6**). IR (neat): *ν* (cm^{-1}) = 2916, 2847, 1737,1462, 1170, 975, 884, 719. HRMS (ESI+) for $C_{30}H_{59}BrO_2$ [M+H]$^+$: *m/z* = 531.3777 (calculated) 531.3766 (observed).

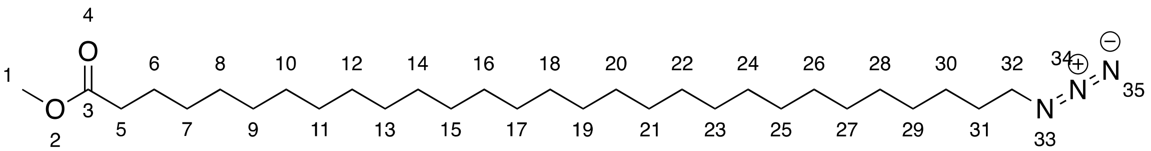

Methyl 29-azidononacosanoate, (az28ME). Methyl 29-bromononacosanoate (**Br28ME (sat.)**) (103 mg, 0.194 mmol, 1 eq), NaN_3 (186mg, 2.862 mmol, 14.7 eq), and 30 mL DMF were added to a 100 mL round bottom flask with a magnetic stir bar. The reaction was stirred at room temperature for 20 h. The product was then extracted with 50 mL ethyl acetate and washed with three additions of 100 mL DI H_2O to remove excess NaN_3. The organic layer was transferred to a round bottom flask and evaporated via roto-evaporation. The remaining solvent was evaporated by blowing a gentle stream of argon gas leaving a pure white solid of **az28ME**. The solid was dried overnight under high vacuum and required no further purification (89 mg, 180mmol, 93% yield). ^{1}H NMR (400 MHz, $CDCl_3$, ppm) δ 3.66 (3H, s, **1**), 3.25 (2H, t, *J*=7.0 Hz, **32**), 2.30 (2H, t, *J*=7.5 Hz, **5**), 1.66 – 1.57 (4H, m, **6, 31**), 1.31 (48H, s, **7, 8, 9, 10, 11, 12, 13, 14, 15, 16, 17, 18, 19, 20, 21, 22, 23, 24, 25, 26, 27, 28, 29, 30**). ^{13}C NMR (101 MHz, $CDCl_3$, ppm) δ 174.5 (**3**), 51.6 (**32**), 51.6 (**1**), 34.3 (**5**), 30.0 – 29.2 (m, **8, 9, 10, 11, 12, 13, 14, 15, 16, 17, 18, 19, 20, 21, 22, 23, 24, 25, 26, 27, 28, 29**), 29.0 (**7**), 26.9 (**31**), 25.1 (**6**). IR (neat): ν (cm^{-1}) = 3047, 2915, 2848, 1740, 1641, 1472, 1462, 1436, 1377, 1169, 1113, 991. HRMS (ESI+) for $C_{30}H_{59}N_3O_2$ $[M+H]^+$: *m/z* = 494.4686 (calculated), 494.4695 (observed).

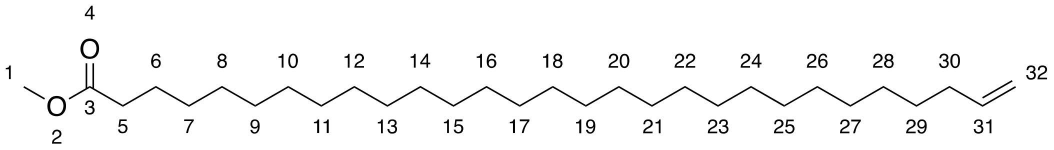

Methyl nonacos-28-enoate, (alkene28ME). $Pd_2(dba)_3$ (21 mg, 22.9 µmol, 0.16 eq.), $[HP(t\text{-}Bu)_2Me)]BF_4$ (21 mg, 72.3 µmol, 0.5 eq.), and a stir bar were added to an oven-dried 50 mL 3-neck flask. The flask was sealed with two rubber septa and a stopcock vacuum adaptor. The flask was then evacuated and backfilled with argon (3 cycles). The flask was detached from the argon line, and 1 mL dioxane (anhydrous), *t*-BuOK (150 µL of a 1 M solution in THF; 150 µmol, 1.0 eq.) and Cy_2NH (130 µL, 717 µmol, 4.9 eq.) were added in turn via syringe. The puncture holes of the septa were sealed with vacuum grease, and the mixture was stirred at r.t. for 2 h. **Br28ME (sat.)** (77 mg, 145 µmol, 1.0 eq.) was placed in a vial with a septum lid. The vial was purged with argon for 30 min. Once the pre-catalyst had reacted for 2 h, **Br28ME (sat.)** was dissolved in 2 mL of anhydrous dioxane, heated until fully dissolved, and transferred into the reaction via a long needle syringe. An additional 2 mL anhydrous dioxane was used to wash the vial and ensure an efficient transfer while maintaining air free conditions. The total reaction volume was 5 mL dioxane. The reaction mixture was stirred at r.t. for 28 h. The reaction solution was vacuumed filtered through a silica plug followed by an additional 100 mL 5% ether in hexanes. This filtration step removed the catalysts from the reaction mixture. The filtrate was transferred to a round bottom flask and evaporated by roto-evaporation. The crude was redissolved in hexanes and purified via silica column chromatography starting with hexanes. The product eluted in 1% ethyl acetate in hexanes. The fractions containing product were collected and combined. The solvent was evaporated using the roto-evaporator and the product was dissolved in hot ethyl acetate and precipitated overnight. The precipitate was filtered leaving a white solid. The solid was collected off the filter by dissolving it in chloroform. The chloroform was evaporated, and the product was dried under high vacuum leaving a white solid. (60 mg, 133 µmol, 92% yield). ^{1}H NMR (400 MHz, $CDCl_3$, ppm) δ 5.81 (1H, ddt, *J*=17.0, 10.0, 7.0 Hz, **31**), 5.16 – 4.77 (2H, m, **32**), 3.66 (3H, s, **1**), 2.30 (2H, t, *J*=7.5 Hz, **5**), 2.04 (2H, q, *J*=7.0 Hz, **30**), 1.66 – 1.58 (2H, m, **6**), 1.41 – 1.34 (2H, m, **29**), 1.25 (44H, s, **7, 8, 9, 10, 11, 12, 13, 14, 15, 16, 17, 18, 19, 20, 21, 22, 23, 24, 25, 26, 27, 28**). ^{13}C NMR (101 MHz, $CDCl_3$, ppm) δ 174.5 (**3**), 139.4 (**31**), 114.2 (**32**), 51.6 (**1**), 34.3 (**5**), 34.0 (**30**), 30.1 – 29.0 (m, **7, 8, 9, 10,**

11, 12, 13, 14, 15, 16, 17, 18, 19, 20, 21, 22, 23, 24, 25, 26, 27, 28, 29), 25.1 (**6**). IR (neat): ν (cm^{-1}) = 3047, 2915, 2848, 1740, 1641, 1472, 1462, 1436, 1377, 1169, 1113, 991. HRMS (ESI+) for $C_{30}H_{58}O_2$ [M+H]$^+$: *m/z* = 451.4515 (calculated), 451.4500 (observed).

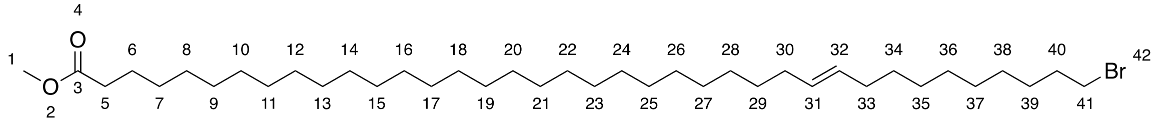

Methyl (*E*)-38-bromooctatriacont-28-enoate, **(Br37ME (unsat.))**. Methyl nonacos-28-enoate (**alkene28ME**) (54 mg, 0.120 mmol, 1 eq), 11-bromoundec-1-ene (560 mg, 2.51 mmol, 20 eq), Grubbs Catalyst 2nd Generation (8 mg, 9.42 μmol, 0.08 eq, 8 mol %), and 10 mL DCM were added to a 25 mL 3-neck round bottom flask containing a magnetic stir bar. A reflux condenser with a vacuum adaptor was connected to the round bottom flask. The remaining necks were sealed with rubber septa and wrapped with parafilm. The round bottom flask was evacuated using a high vacuum and backfilled with argon gas. The round bottom flask was then subjected to three freeze-thaw cycles to remove dissolved oxygen. While freezing, the flask was evacuated. While thawing, the flask was backfilled with argon gas. Once completely thawed, the flask was heated up to 40 °C and refluxed overnight for 28 h. The flask was cooled to r.t., the solvent was evaporated, and the contents of the round bottom flask were dried under high vacuum for 3 h to remove residual DCM, leaving behind an oily mixture. The mixture was dissolved in a minimal amount of hexanes, a dark precipitate crashed out, and the suspended solution was loaded onto a SiO_2 column prepared with a hexane mobile phase. The 11-bromoundec-1-ene starting material and symmetric side product were eluted with 200 mL hexanes into a single fraction. The mobile phase polarity was increased to 2% ethyl acetate in hexanes and 10 mL fractions were collected. A faint yellow band containing the product could be visualized moving down the column. Afterwards, the mobile phase was switched to DCM to collect any remaining material. The fractions containing product were collected and reduced in volume via roto-evaporation leaving behind a clear oil. The remaining solvent was removed using a stream of argon gas leaving behind a white waxy solid. The solid was then dried *in vacuo* (44 mg, 0.062 mmol, 56% yield). ^{1}H NMR (400 MHz, $CDCl_3$, ppm) δ 5.47 – 5.26 (2H, m, **31, 32**), 3.66 (3H, s, **1**), 3.40 (2H, t, *J*=7.0 Hz, **41**), 2.30 (2H, t, *J*=7.5 Hz, **5**), 2.08 – 1.92 (4H, m, **30, 33**), 1.91 – 1.78 (2H, m, **40**), 1.66 – 1.59 (2H, m, **6**), 1.48 – 1.14 (58H, m, **7, 8, 9, 10, 11, 12, 13, 14, 15, 16, 17, 18, 19, 20, 21, 22, 23, 24, 25, 26, 27, 28, 29, 34, 35, 36, 37, 38, 39**). ^{13}C NMR (101 MHz, $CDCl_3$, ppm) δ 174.5 (**3**), 130.6 (**31**), 130.4 (**32**), 51.6 (**1**), 34.3 (**5**), 34.2 (**41**), 33.0 (**40**), 32.9 – 32.4 (m, **30, 33**), 30.3 – 29.1 (m, **7, 8, 9, 10, 11, 12, 13, 14, 15, 16, 17, 18, 19, 20, 21, 22, 23, 24, 25, 26, 27, 28, 29, 34, 35, 36, 37, 38**), 28.3 (**39**), 25.1 (**6**). IR (neat): ν (cm^{-1}) = 3043, 2915, 2847, 1739, 1472, 1462, 1365, 1167, 964, 882, 804. HRMS (ESI+) for $C_{39}H_{75}BrO_2$ [M+Na]$^+$: *m/z* = 679.4835 (calculated), 679.4682 (observed).

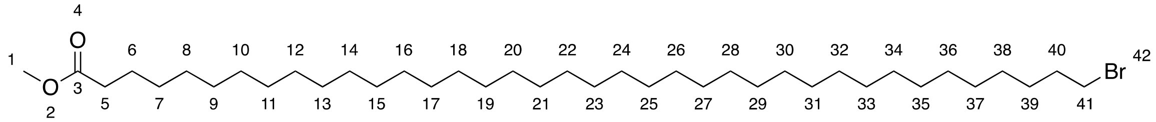

Methyl 38-bromooctatriacontanoate, (Br37ME (sat.)). Methyl (*E*)-38-bromooctatriacont-28-enoate (**Br37ME (unsat.)**) (37 mg, 0.056 mmol.) was added to a 100 mL round bottom flask containing a magnetic stirrer. The solid compound was dissolved in 30 mL of 1:2 MeOH:ethyl acetate. A small scoop of Pd/C (10 wt. % loading) was added to the solution and the round bottom flask was sealed with a stopcock vacuum adaptor. The flask was evacuated and backfilled with argon gas for three cycles. On the final cycle, the flask was evacuated, and a soccer ball pressurized with hydrogen gas was attached to the flask. The reaction stirred at room temperature under the hydrogen atmosphere for 18 h. The soccer ball was removed, and the solution was gravity filtered through a celite plug using hexanes. The filtrate was collected, and the solvent removed via roto-evaporation. The crude product was recrystallized by dissolving

in 1 mL chloroform and precipitated using a combination of ethyl acetate and methanol. The precipitate was then redissolved by heating the flask to 50 °C in a water bath. Once redissolved, the flask was removed from the water bath and the precipitate slowly crashed out of solution at room temperature. The precipitate was vacuum filtered off using a fine glass frit Buchner funnel, washed with methanol, and collected off the frit by dissolving the solid in chloroform. The chloroform solution was reduced in volume leaving behind a clear oil. The remaining solvent was removed under a gentle stream of argon gas leaving behind a white waxy solid. The solid was then dried *in vacuo* at r.t. (17 mg, 0.025 mmol, 46% yield). ^{1}H NMR (400 MHz, $CDCl_3$, ppm) δ 3.66 (3H, s, **1**), 3.40 (2H, t, *J*=7.0 Hz, **41**), 2.30 (2H, t, *J*=7.5 Hz, **5**), 1.85 (2H, p, *J*=7.0 Hz, **40**), 1.66 – 1.55 (2H, m, **6**), 1.48 – 1.36 (2H, m, **39**), 1.25 (64H, s, **7, 8, 9, 10, 11, 13, 14, 15, 16, 17, 18, 19, 20, 21, 22, 23, 24, 25, 26, 27, 28, 29, 30, 31, 32, 33, 34, 35, 36, 37, 38**). ^{13}C NMR (101 MHz, $CDCl_3$, ppm) δ 174.5 (**3**), 51.6 (**1**), 34.3 (**5**), 34.2 (**41**), 33.0 (**40**), 30.1 – 28.8 (m, **7, 8, 9, 10, 11, 12, 13, 14, 15, 16, 17, 18, 19, 20, 21, 22, 23, 24, 25, 26, 27, 28, 29, 30, 31, 32, 33, 34, 35, 36, 37, 38**), 28.3 (**39**), 25.1 (**6**). IR (neat): ν (cm^{-1}) = 2916, 2848, 1738, 1473, 1461, 1261, 1169, 1097, 1018.

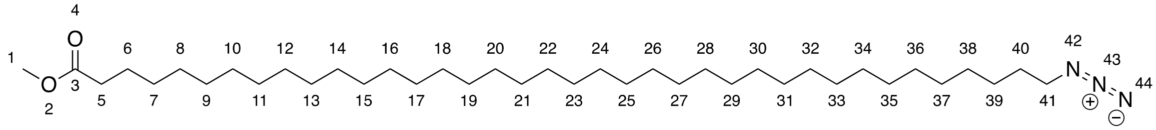

Methyl 38-azidooctatriacontanoate, (az37ME). Methyl 38-bromooctatriacontanoate (**Br37ME (sat.)**) (10 mg, 16 µmol, 1 eq.) and NaN_3 (74 mg, 1.14 mmol, 71 eq) were added to a 50 mL round bottom flask with a stir bar. The reagents were suspended in 5 mL toluene and 5 mL DMF, the flask stoppered with a rubber septum, and stirred at room temperature for 72 h. The reaction was monitored for completion using NMR. After 72 h, the solution was transferred to a 125 mL extraction funnel. 50 mL of DI H_2O was then added to the extraction funnel. 50 mL of ethyl acetate was used to wash the round bottom flask ensuring an efficient transfer and added to the extraction funnel. The extraction funnel was shaken, and the aqueous layer was collected. The organic layer was washed 2 more times with 50 mL DI water to remove any remaining DMF. The organic layer was collected in a pre-weighed 100 mL round bottom flask and the solvent was removed via roto-evaporation, followed by a gentle stream of argon gas, and left to dry overnight under high vacuum. The product was a white, waxy solid that required no further purification (9 mg, 15 µmol, 95% yield). ^{1}H NMR (400 MHz, $CDCl_3$, ppm) δ 3.66 (3H, s, **1**), 3.25 (2H, t, *J*=7.0 Hz, **41**), 2.30 (2H, t, *J*=7.5 Hz, **5**), 1.66 – 1.55 (4H, m, **6, 40**), 1.25 (66H, s, **7, 8, 9, 10, 11, 12, 13, 14, 15, 16, 17, 18, 19, 20, 21, 22, 23, 25, 26, 27, 28, 29, 30, 31, 32, 33, 34, 35, 36, 37, 38, 39**). ^{13}C NMR (101 MHz, $CDCl_3$, ppm) δ 174.5 (**3**), 51.7 (**41**), 51.6 (**1**), 34.3 (**5**), 30.4 – 29.1 (m, **8, 9, 10, 11, 12, 13, 14, 15, 16, 17, 18, 19, 20, 21, 22, 23, 24, 25, 26, 27, 28, 29, 30, 31, 32, 33, 34, 35, 36, 37, 38, 39**), 29.0 (**7**), 26.9 (**40**), 25.1 (**6**). IR (neat): ν (cm^{-1}) = 2916, 2848, 2096, 1472, 1462, 1365, 1143, 1094, 1018, 883, 801.

S1.4. ^{1}H and ^{13}C NMR spectra

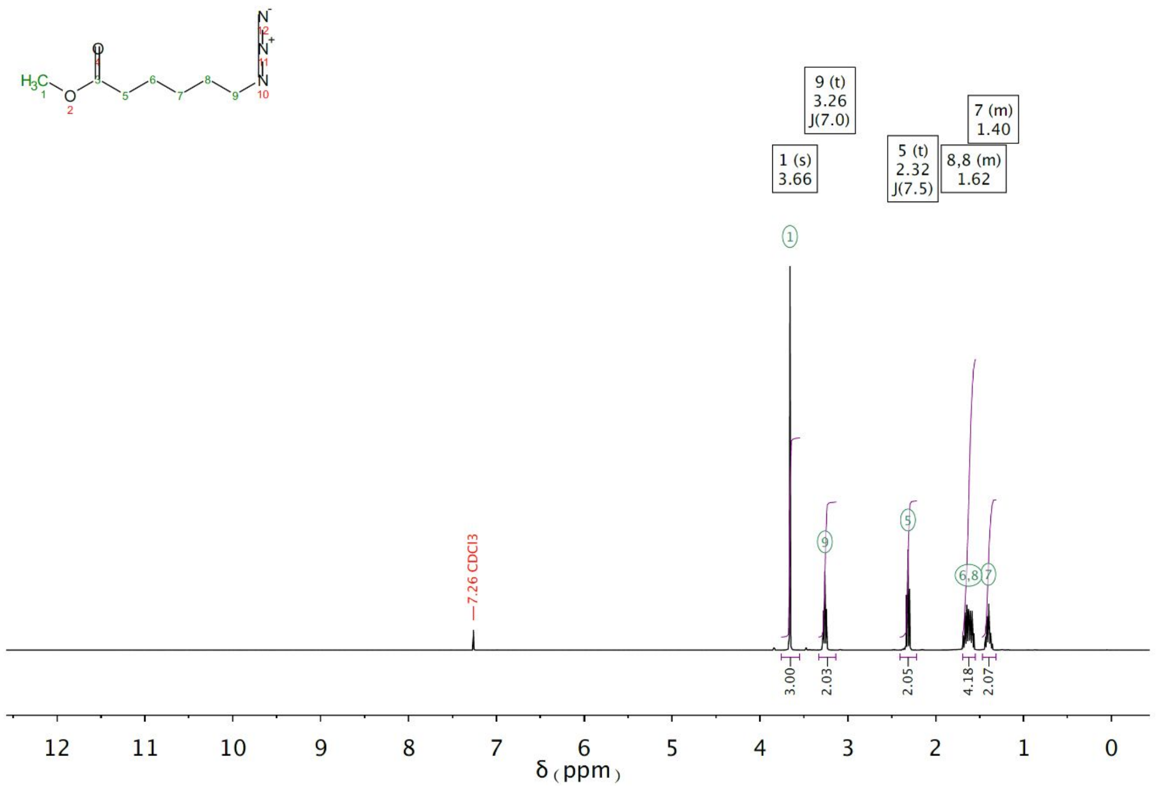

Figure S1. ^{1}H NMR (400 MHz) spectrum of **az5ME** in $CDCl_3$ at 298 K.

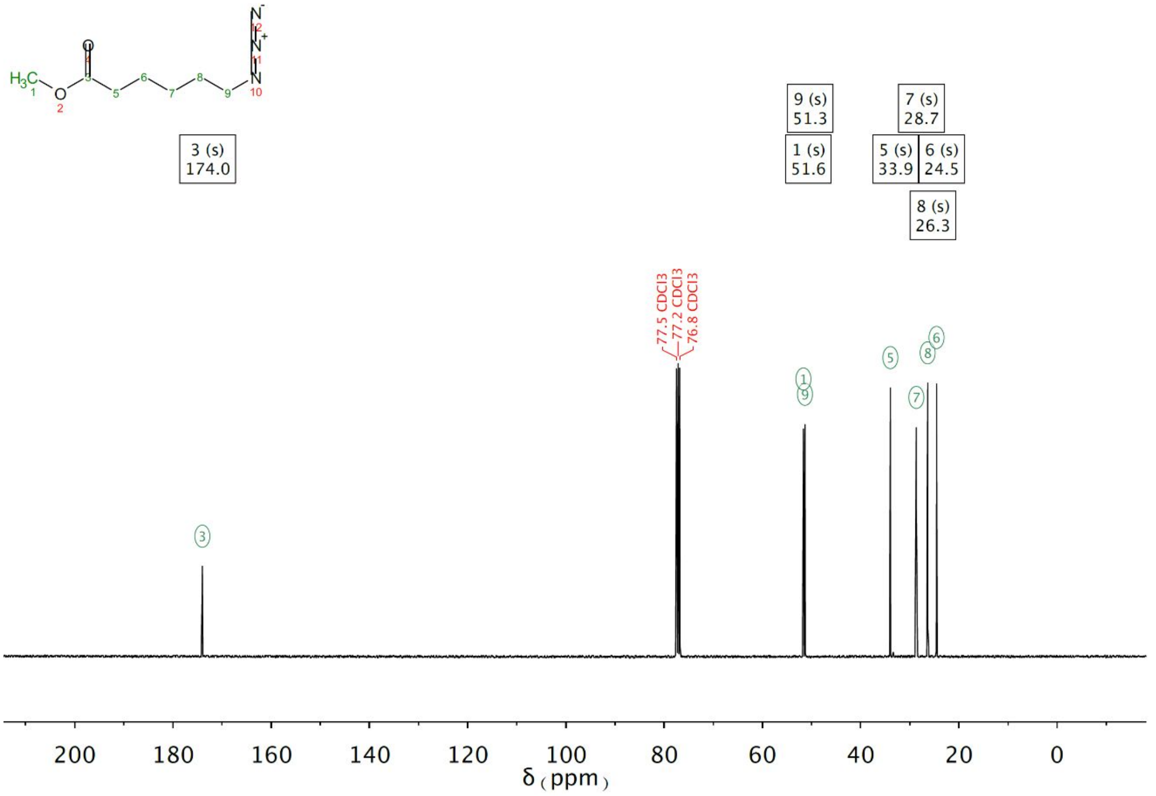

Figure S2. ^{13}C NMR (101 MHz) spectrum of **az5ME** in $CDCl_3$ at 298 K.

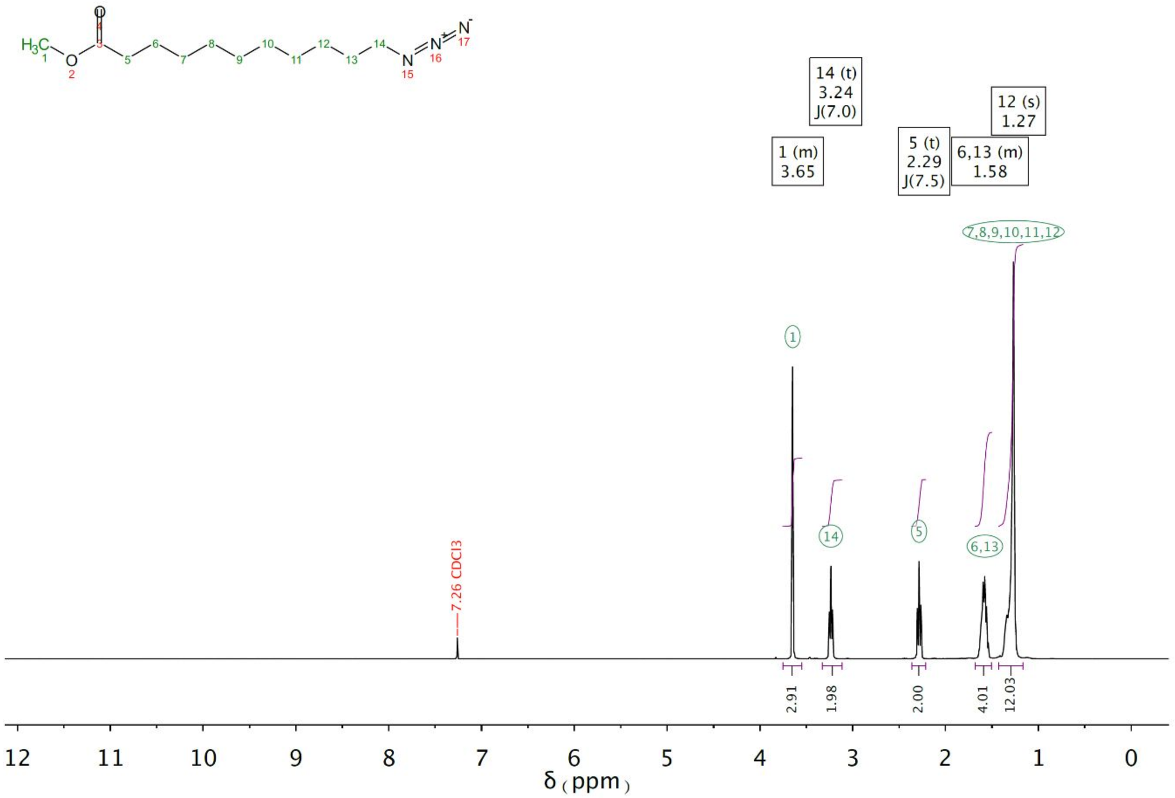

Figure S3. ^{1}H NMR (400 MHz) spectrum of **az10ME** in $CDCl_3$ at 298 K.

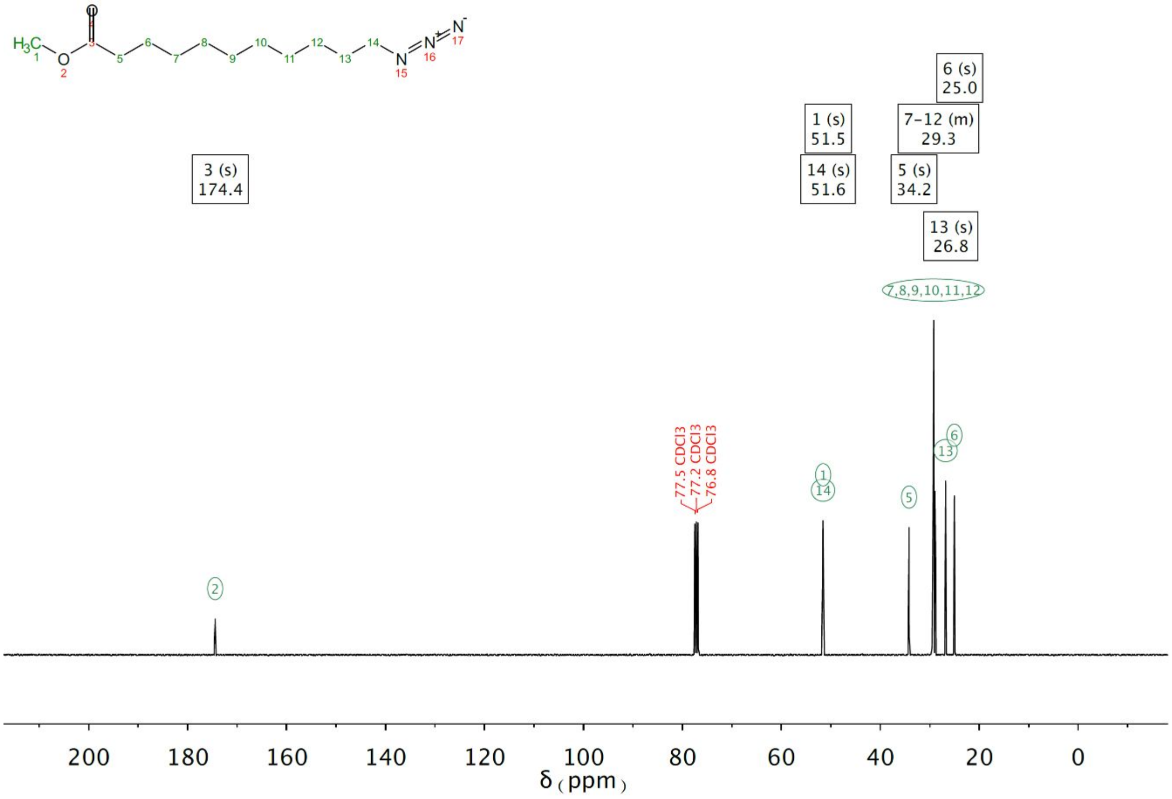

Figure S4. ^{13}C NMR (101 MHz) spectrum of **az10OH** in $CDCl_3$ at 298 K.

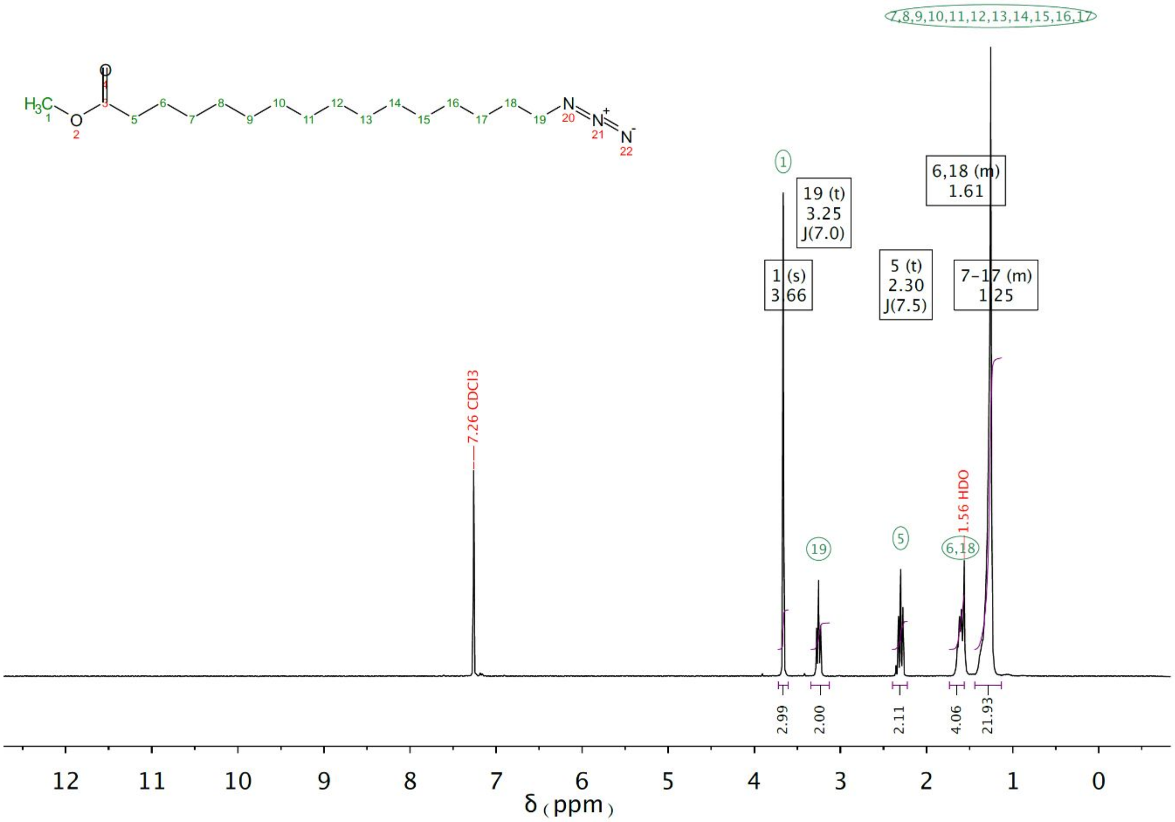

Figure S5. ^{1}H NMR (400 MHz) spectrum of **az15ME** in $CDCl_3$ at 298 K.

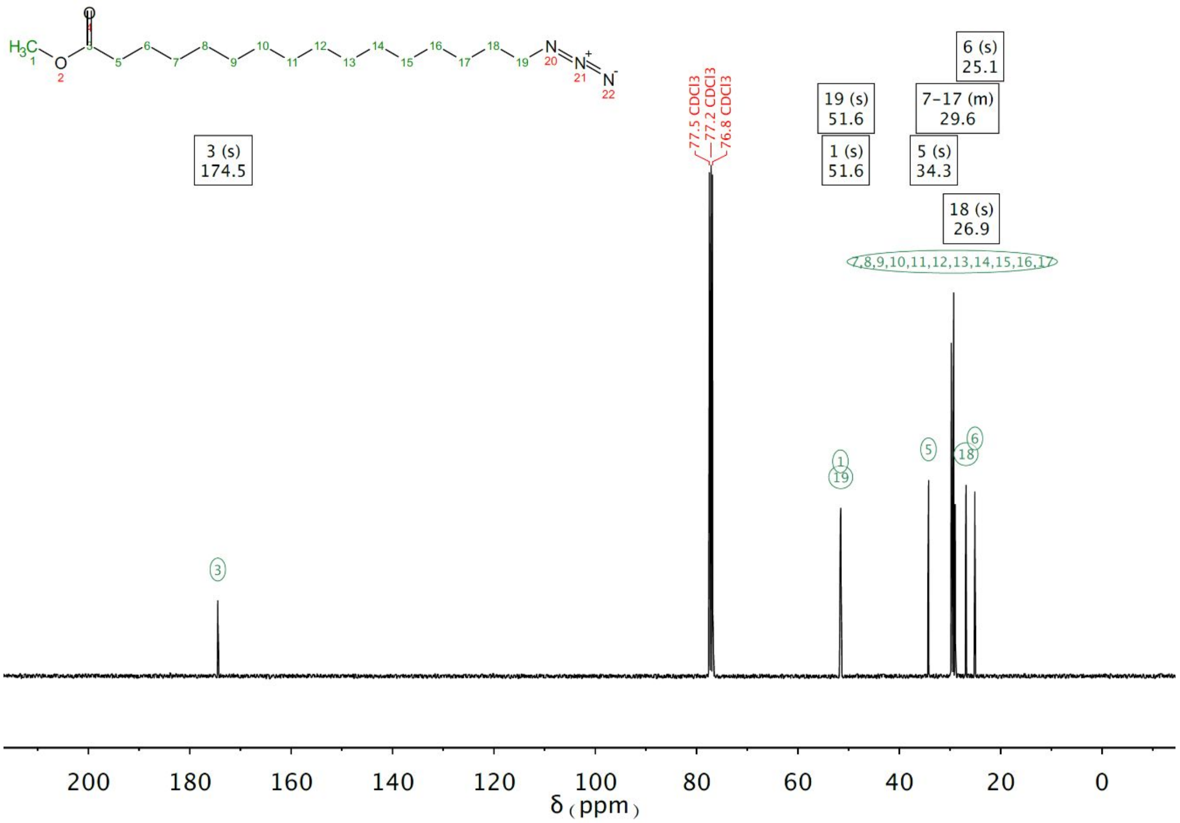

Figure S6. ^{13}C NMR (101 MHz) spectrum of **az15ME** in $CDCl_3$ at 298 K.

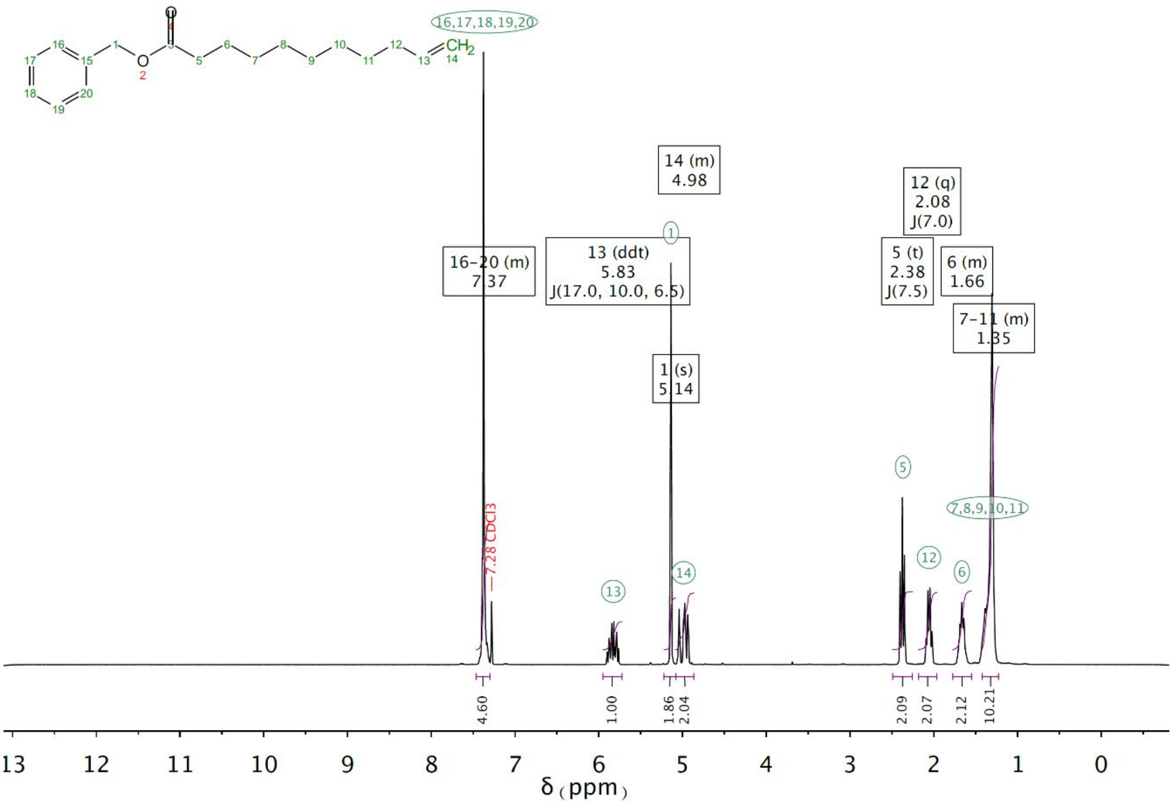

Figure S7. ^{1}H NMR (300 MHz) spectrum of **alkene10BN** in $CDCl_3$ at 298 K.

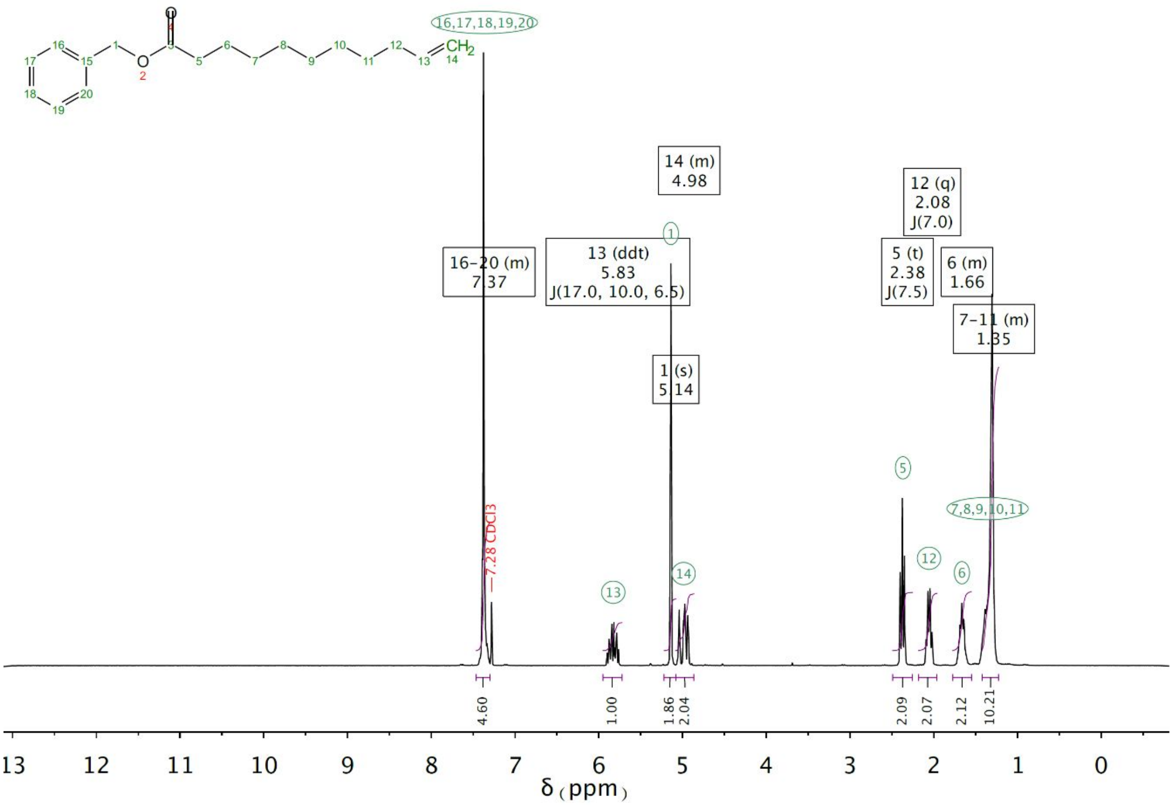

Figure S8. ^{13}C NMR (101 MHz) spectrum of **alkene10BN** in $CDCl_3$ at 298 K.

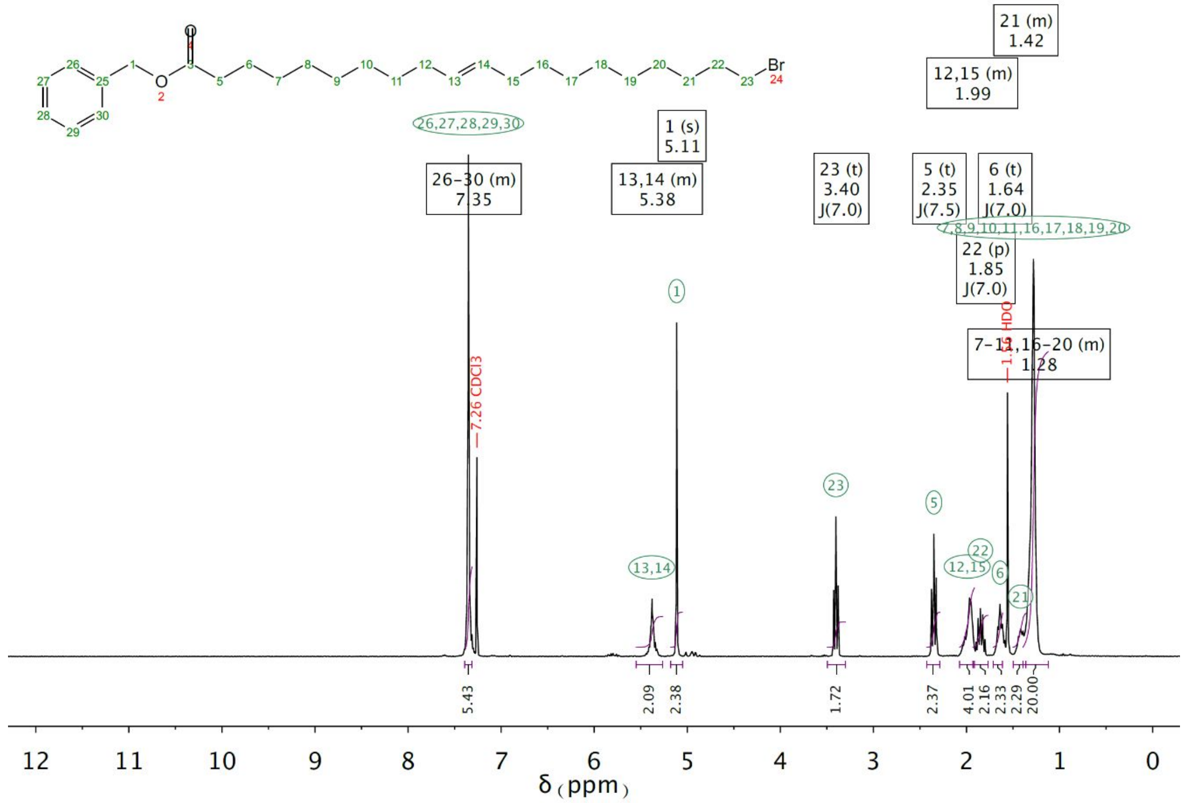

Figure S9. ^{1}H NMR (300 MHz) spectrum of **Br19BN** in $CDCl_3$ at 298 K.

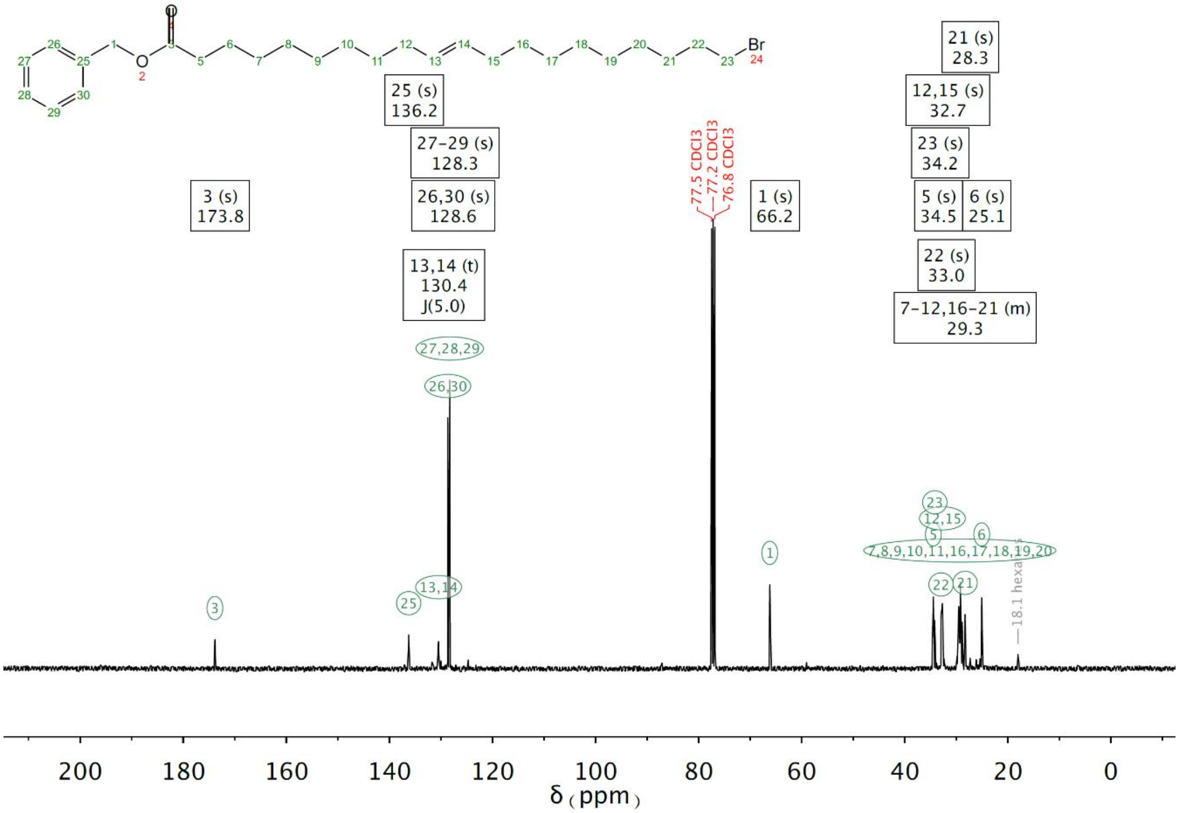

Figure S10. ^{13}C NMR (101 MHz) spectrum of **Br19BN** in $CDCl_3$ at 298 K.

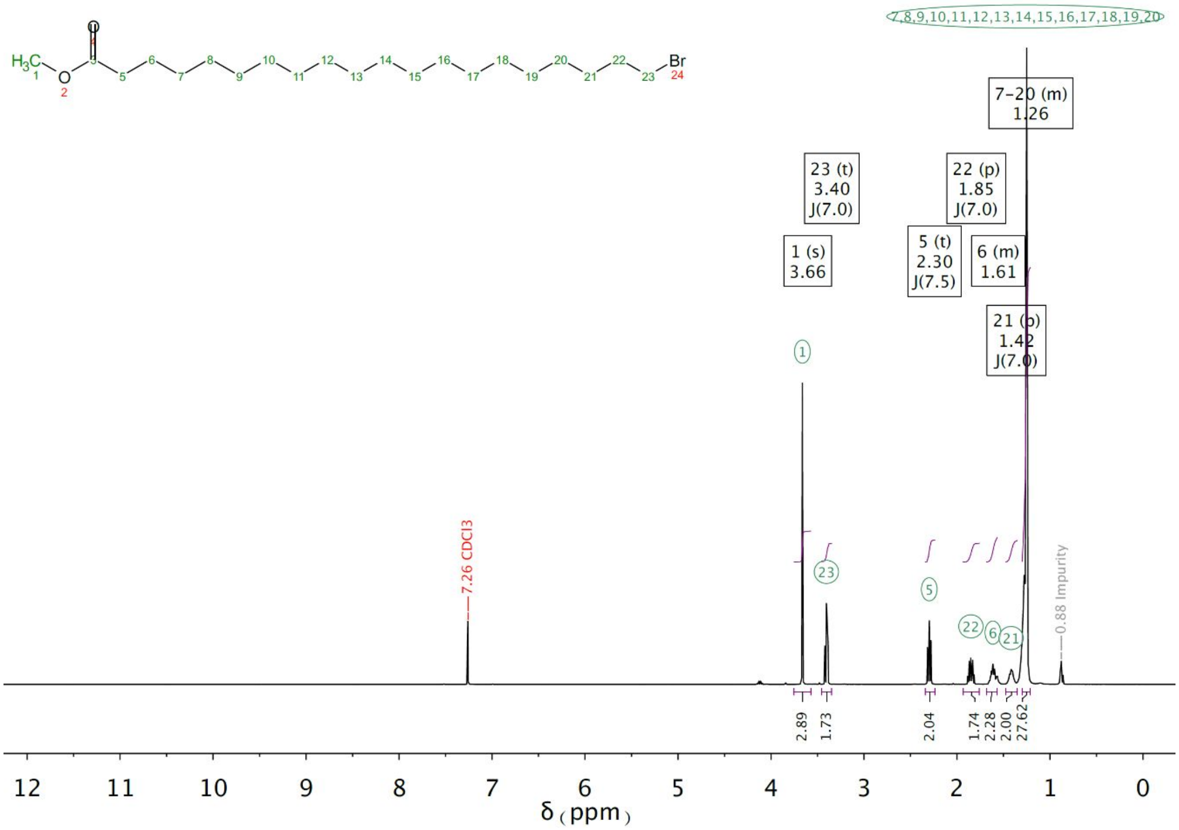

Figure S11. ^{1}H NMR (400 MHz) spectrum of **Br19ME** in $CDCl_3$ at 298 K.

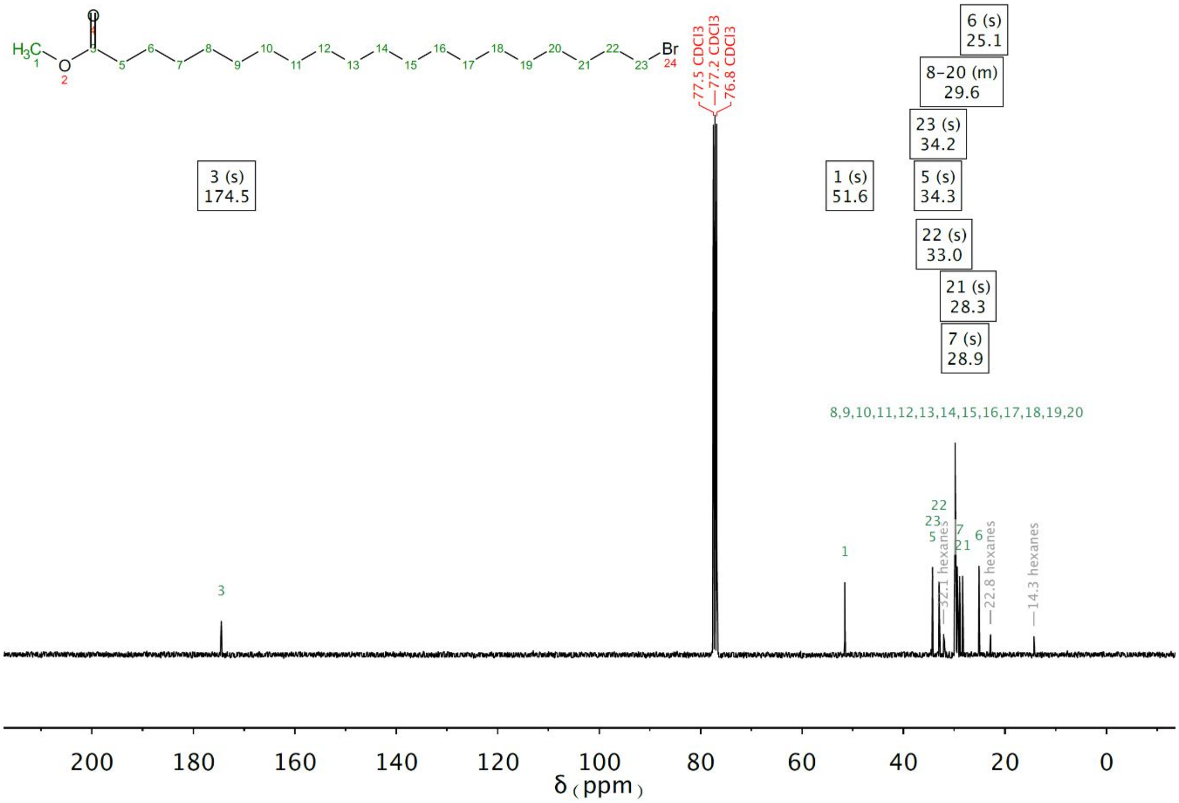

Figure S12. ^{13}C NMR (101 MHz) spectrum of **Br19ME** in $CDCl_3$ at 298 K.

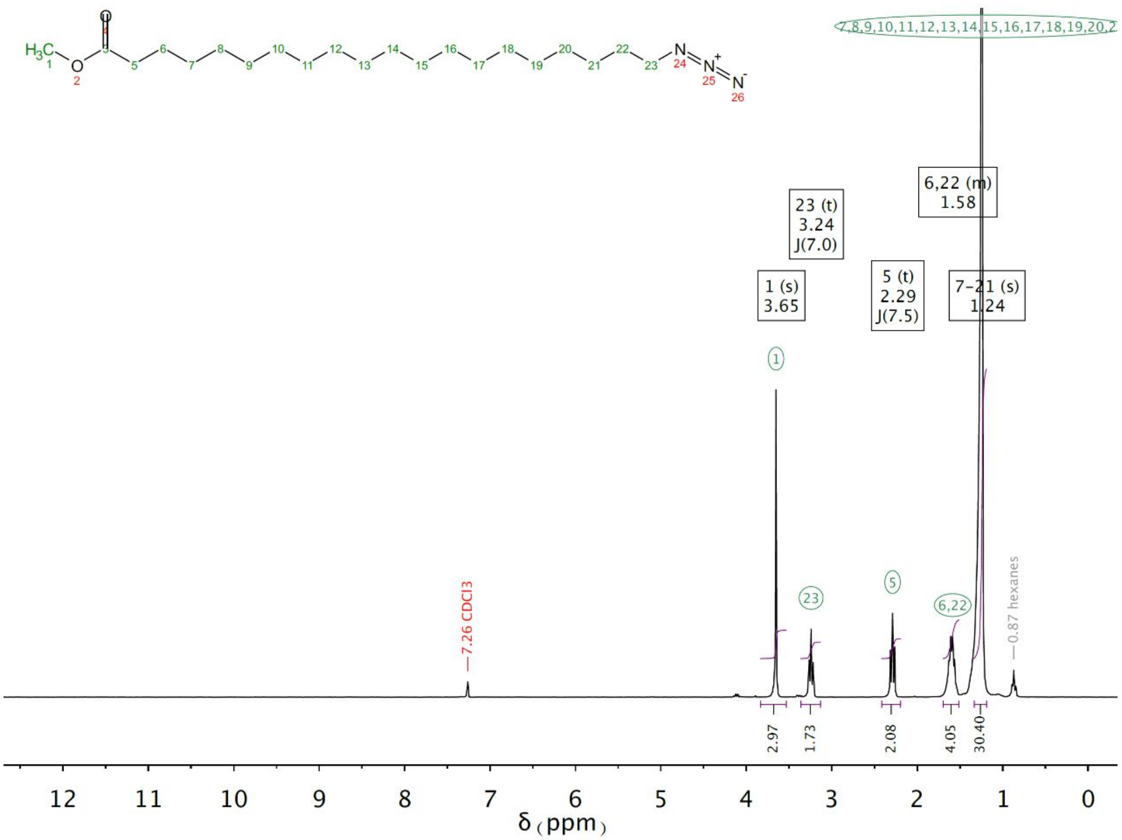

Figure S13. ^{1}H NMR (300 MHz) spectrum of **az19ME** in $CDCl_3$ at 298 K.

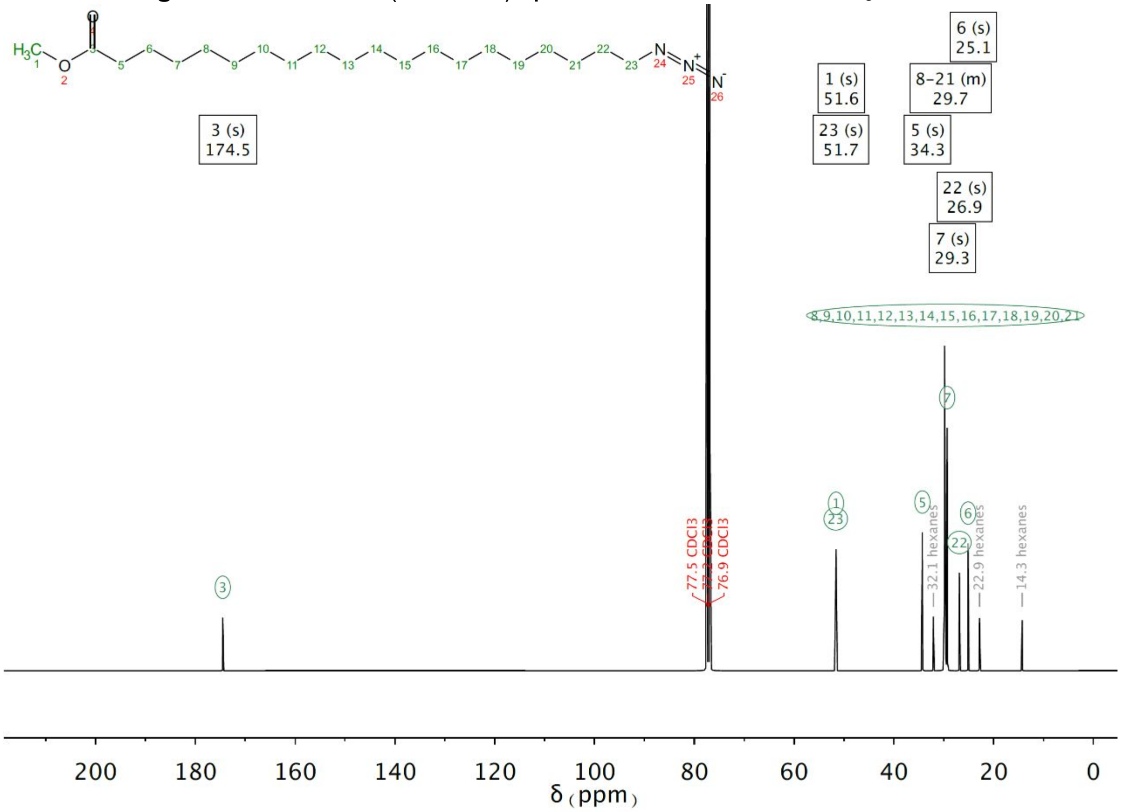

Figure S14. ^{13}C NMR (101 MHz) spectrum of **az19ME** in $CDCl_3$ at 298 K.

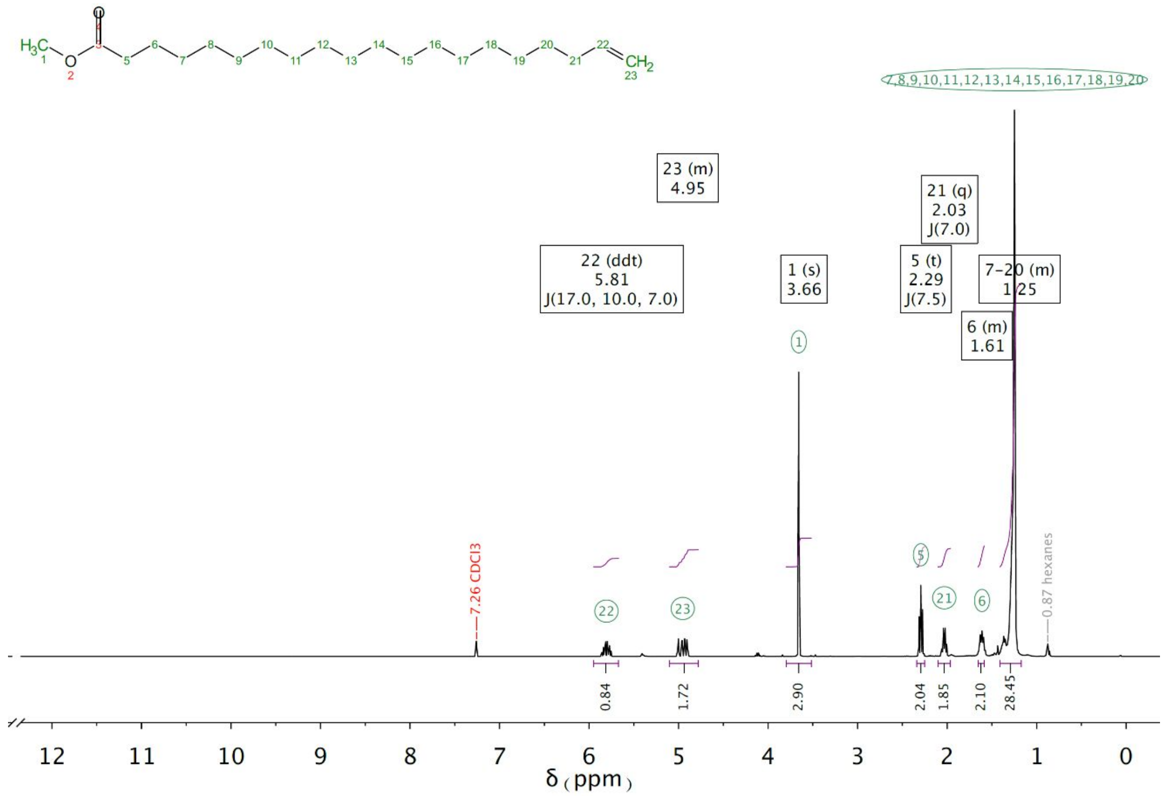

Figure S15. ^{1}H NMR (400 MHz) spectrum of **alkene19ME** in $CDCl_3$ at 298 K.

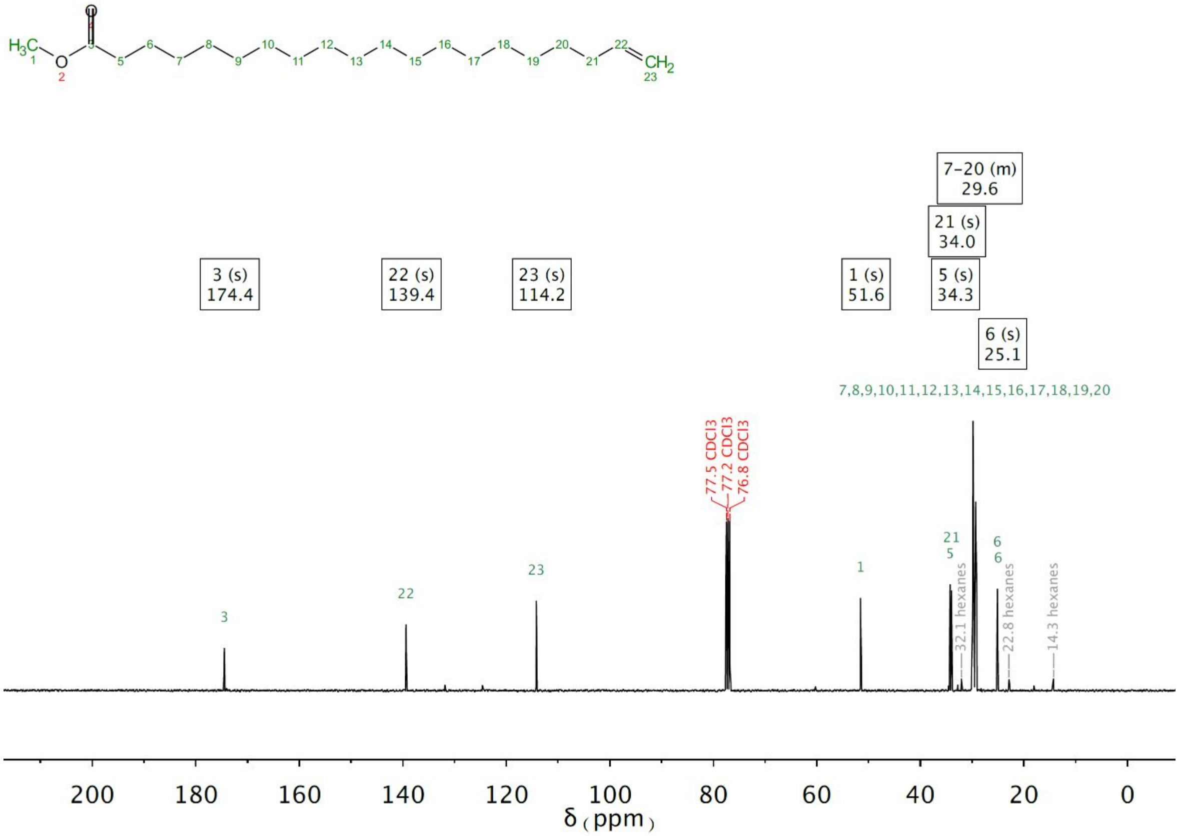

Figure S16. ^{13}C NMR (101 MHz) spectrum of **alkene19ME** in $CDCl_3$ at 298 K.

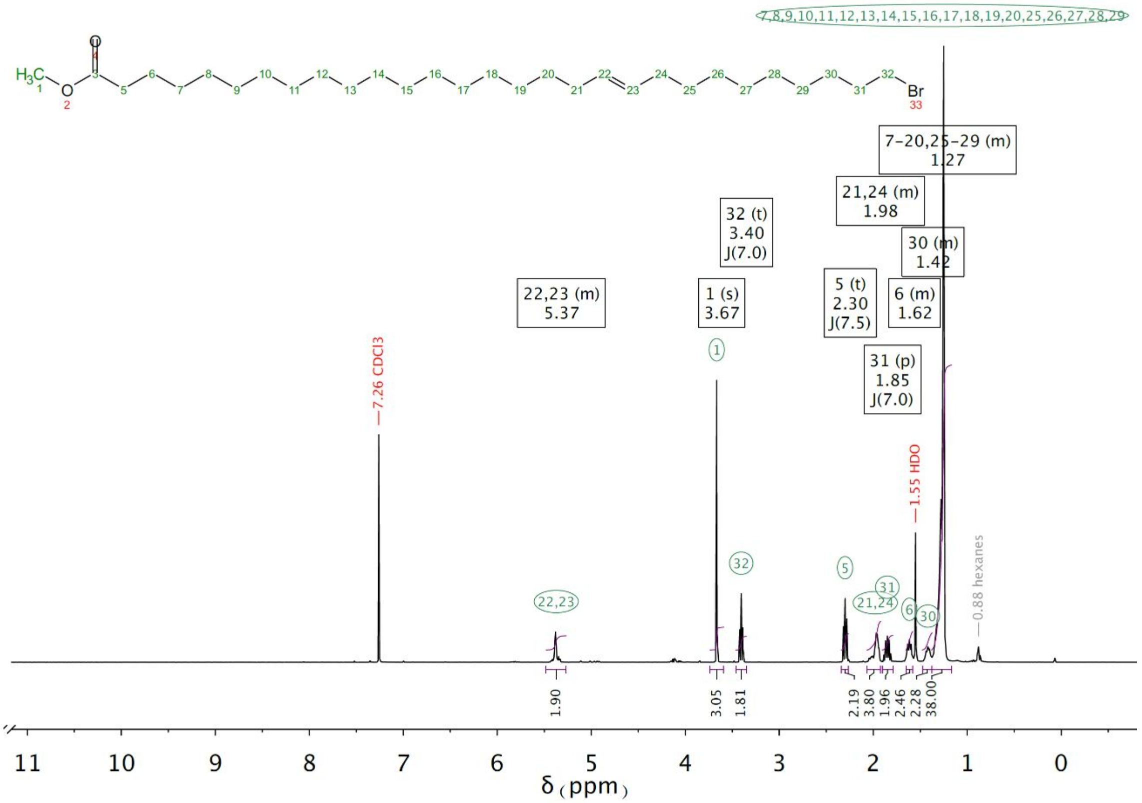

Figure S17. ^{1}H NMR (400 MHz) spectrum of **Br28ME (unsat.)** in $CDCl_3$ at 298 K.

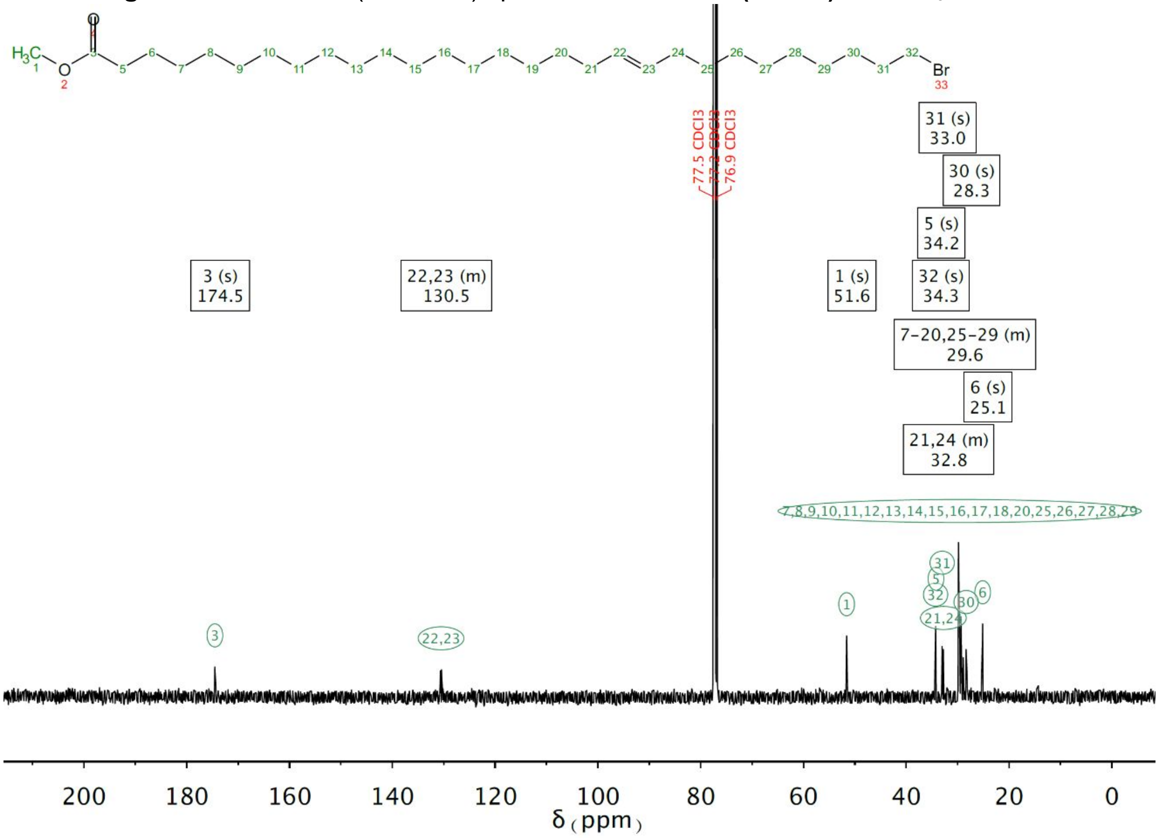

Figure S18. ^{13}C NMR (101 MHz) spectrum of **Br28ME (unsat.)** in $CDCl_3$ at 298 K.

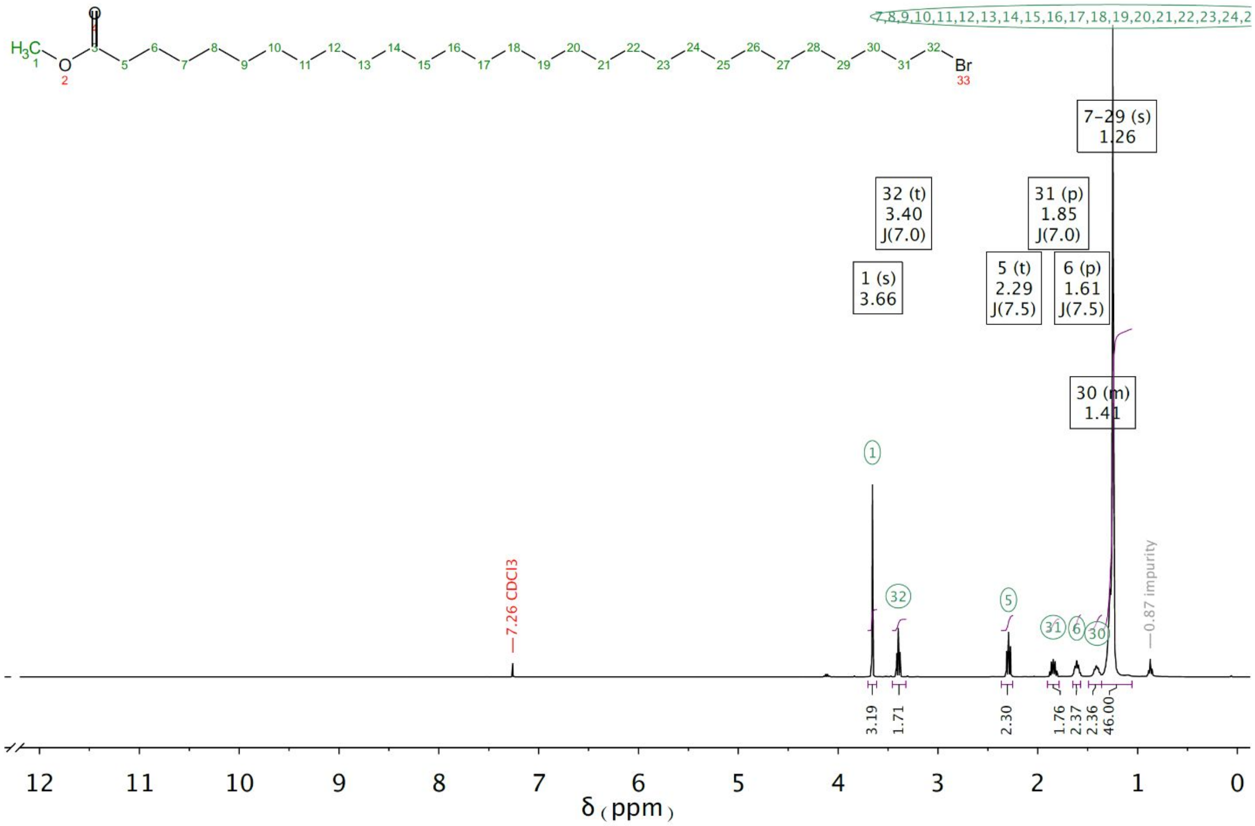

Figure S19. ^{1}H NMR (400 MHz) spectrum of **Br28ME (sat.)** in $CDCl_3$ at 298 K.

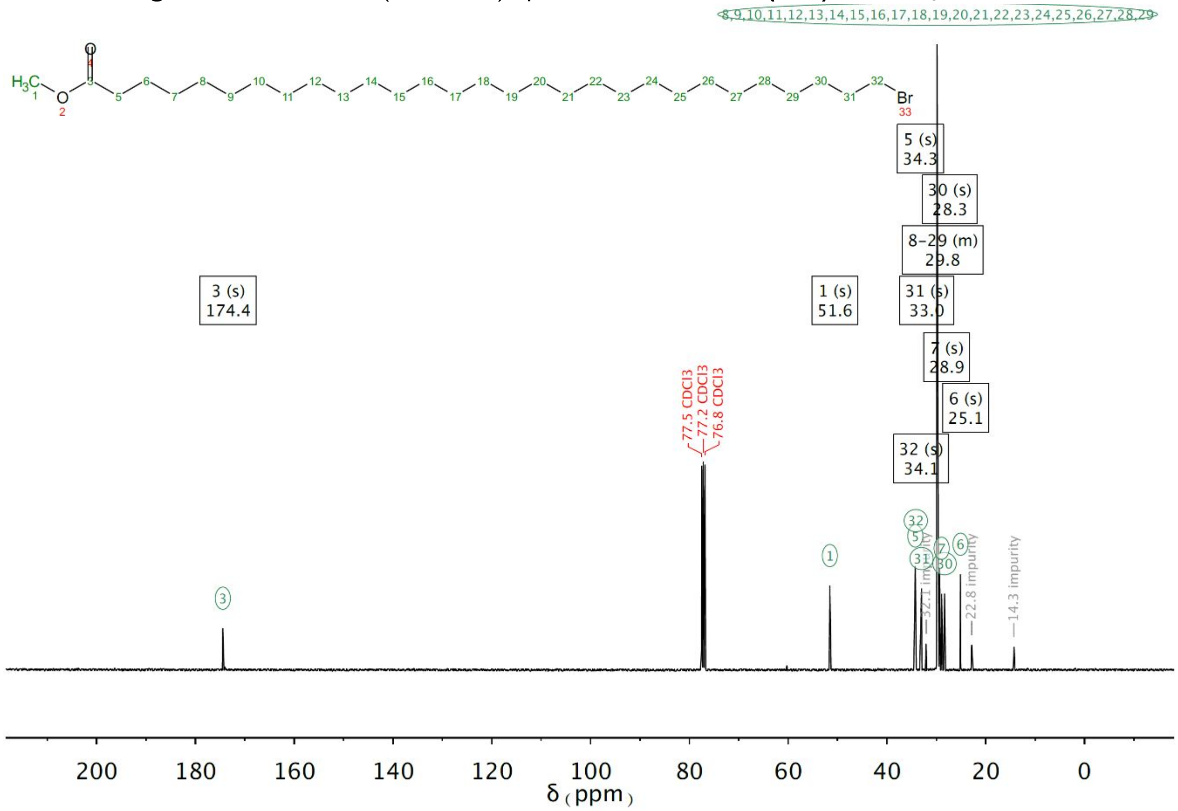

Figure S20. ^{13}C NMR (101 MHz) spectrum of **Br28ME (sat.)** in $CDCl_3$ at 298 K.

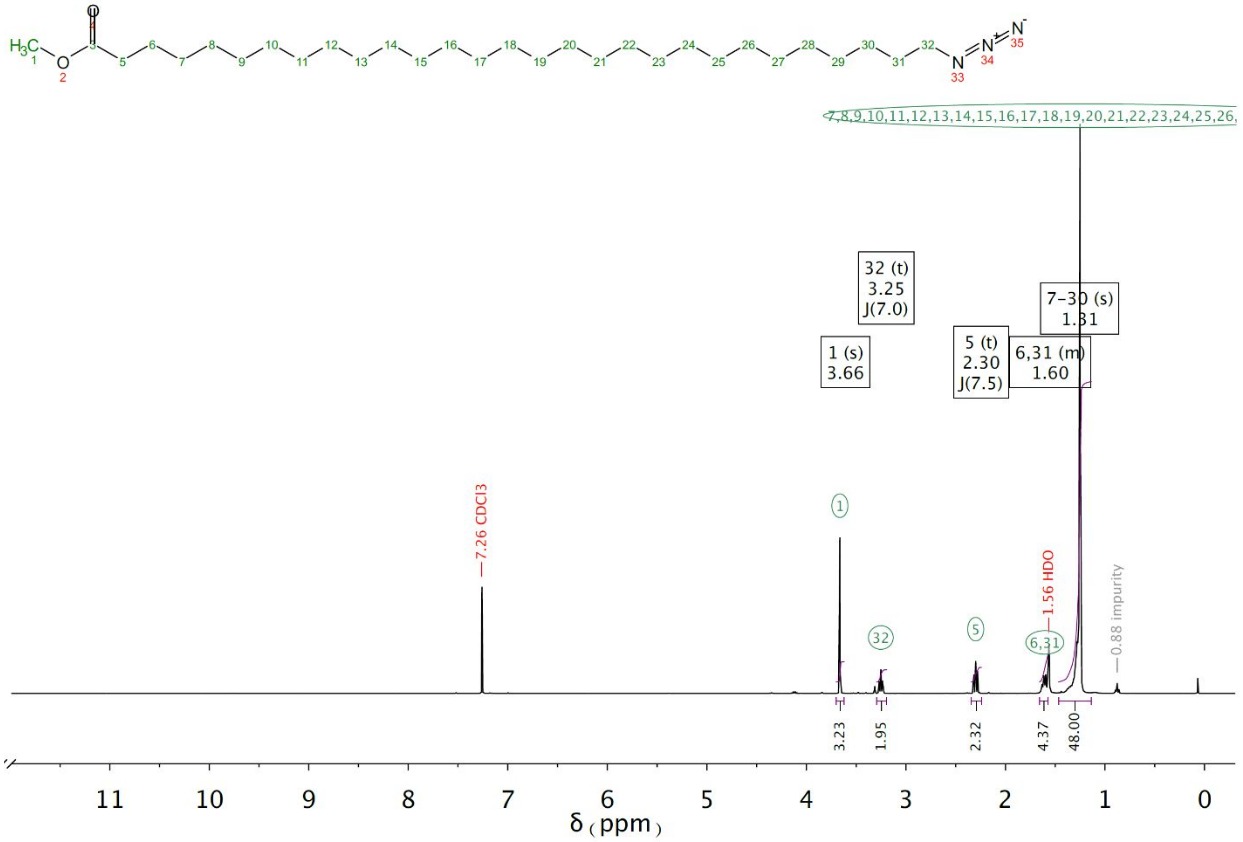

Figure S221. ^{1}H NMR (400 MHz) spectrum of **az28ME** in $CDCl_3$ at 298 K.

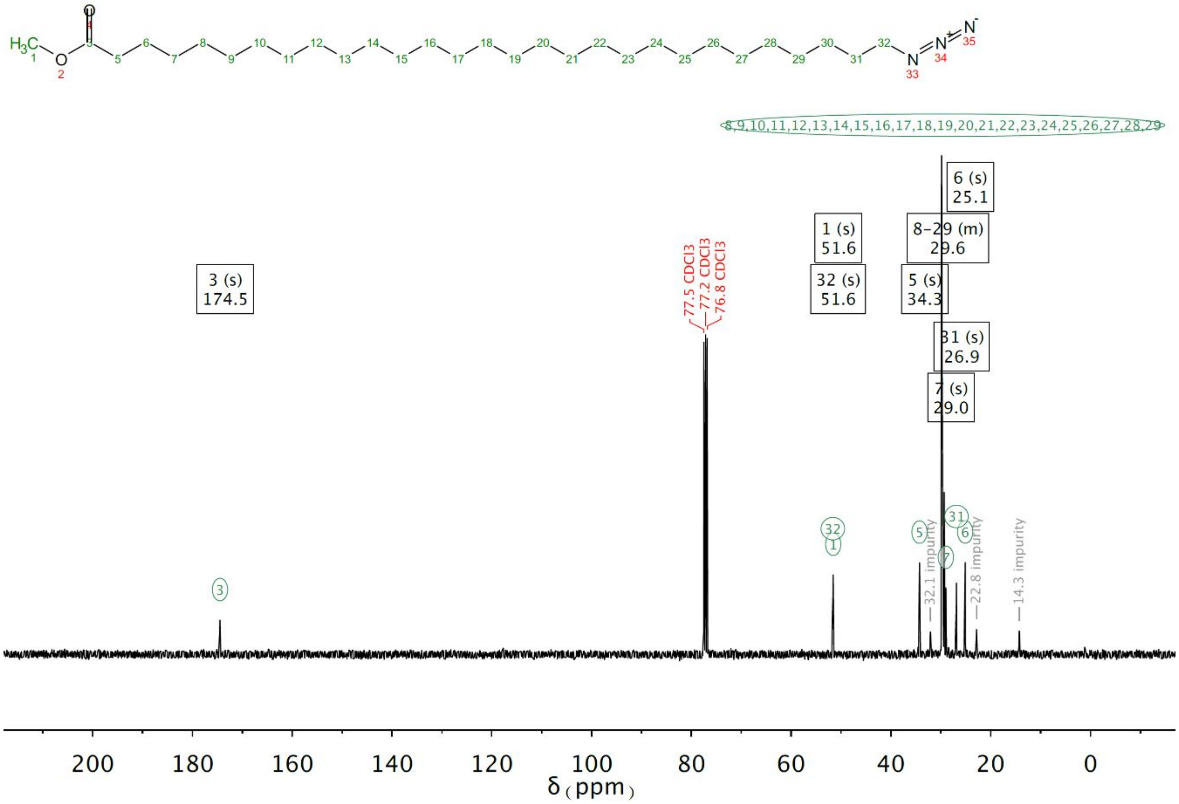

Figure S22. ^{13}C NMR (101 MHz) spectrum of **az28ME** in $CDCl_3$ at 298 K.

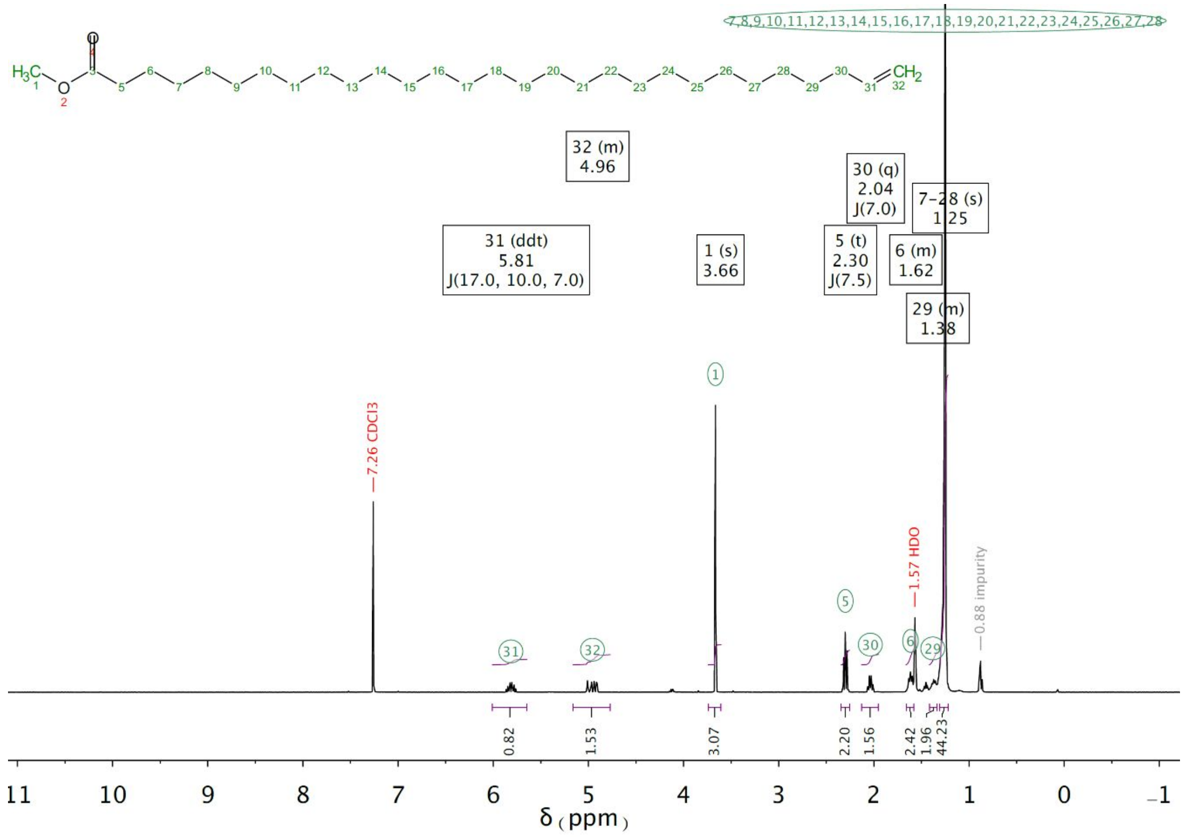

Figure S222. ^{1}H NMR (400 MHz) spectrum of **alkene28ME** in $CDCl_3$ at 298 K.

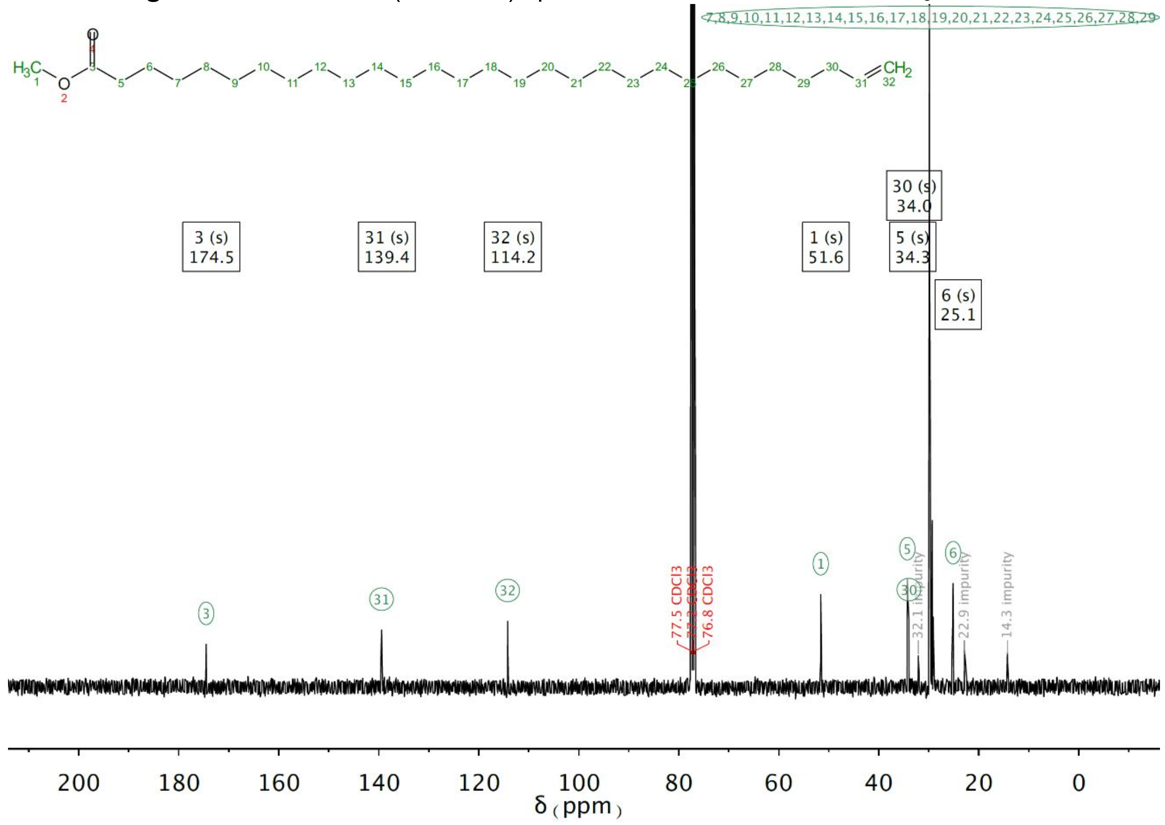

Figure S24. ^{13}C NMR (101 MHz) spectrum of **alkene28ME** in $CDCl_3$ at 298 K.

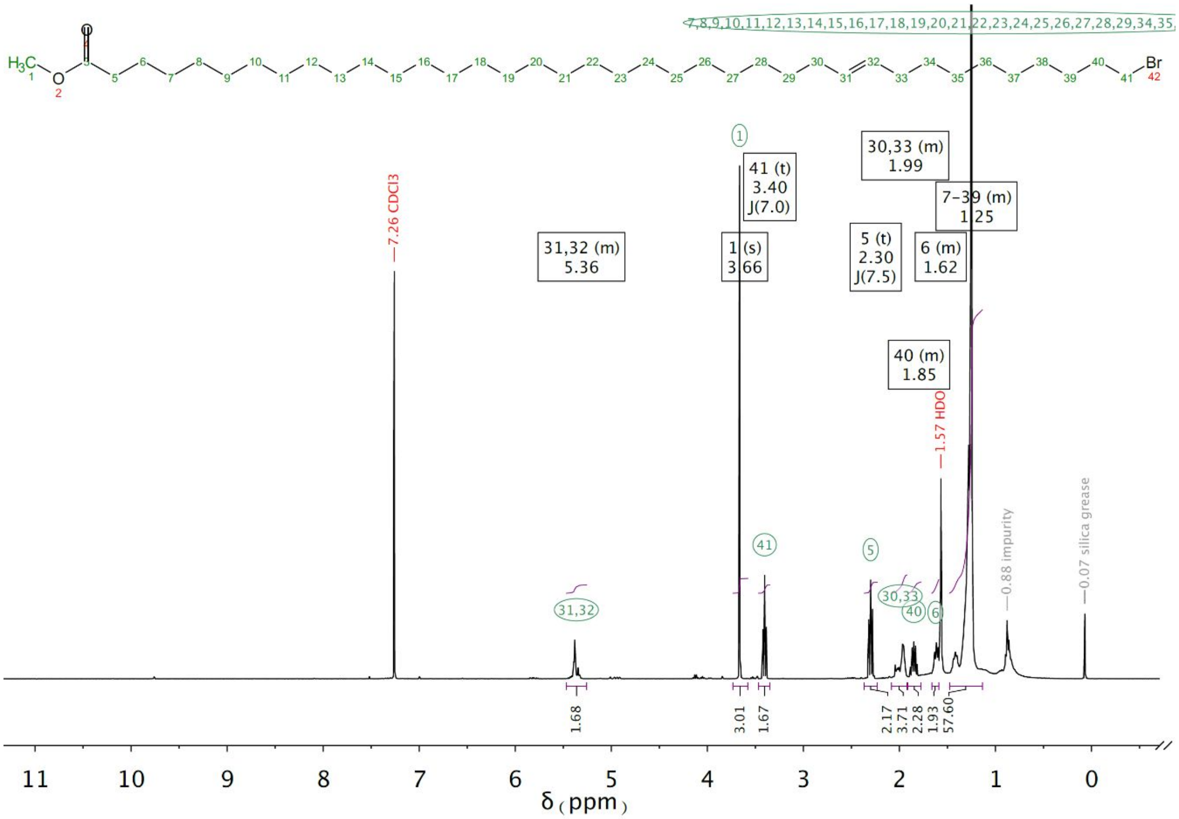

Figure S25. ^{1}H NMR (400 MHz) spectrum of **Br37ME (unsat.)** in $CDCl_3$ at 298 K.

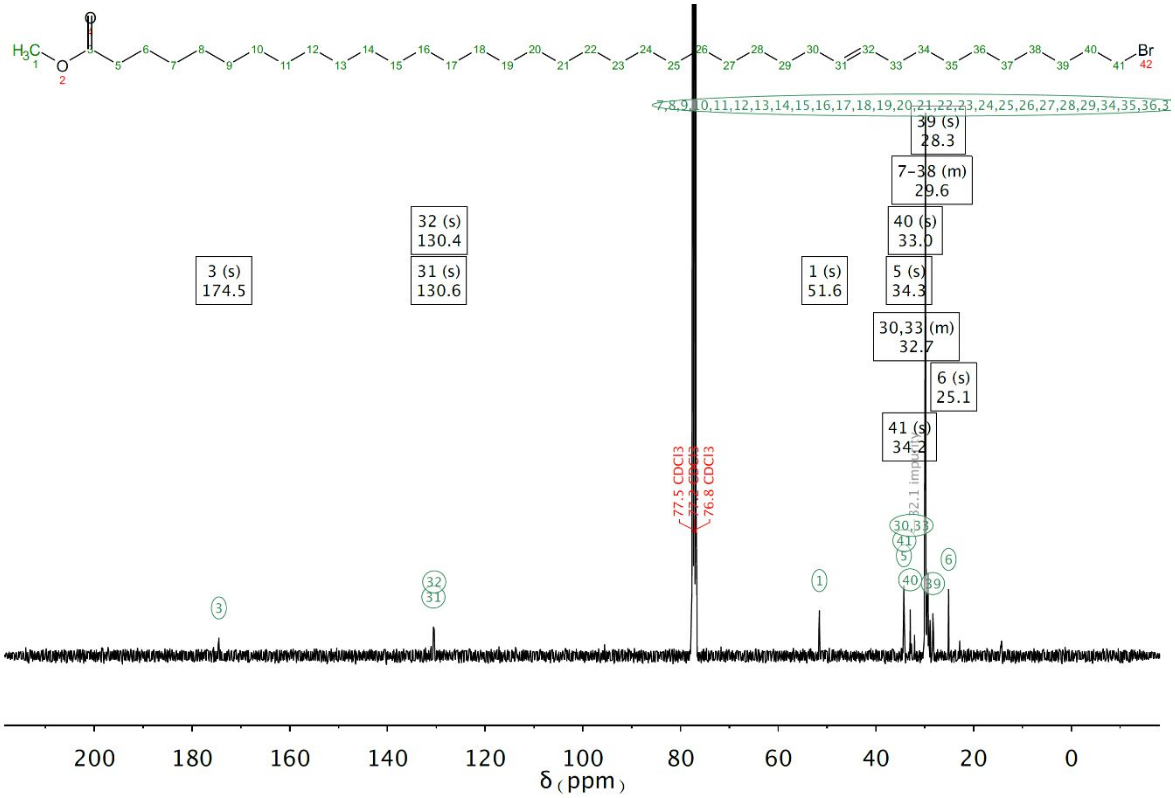

Figure S26. ^{13}C NMR (101 MHz) spectrum of **Br37ME (unsat.)** in $CDCl_3$ at 298 K.

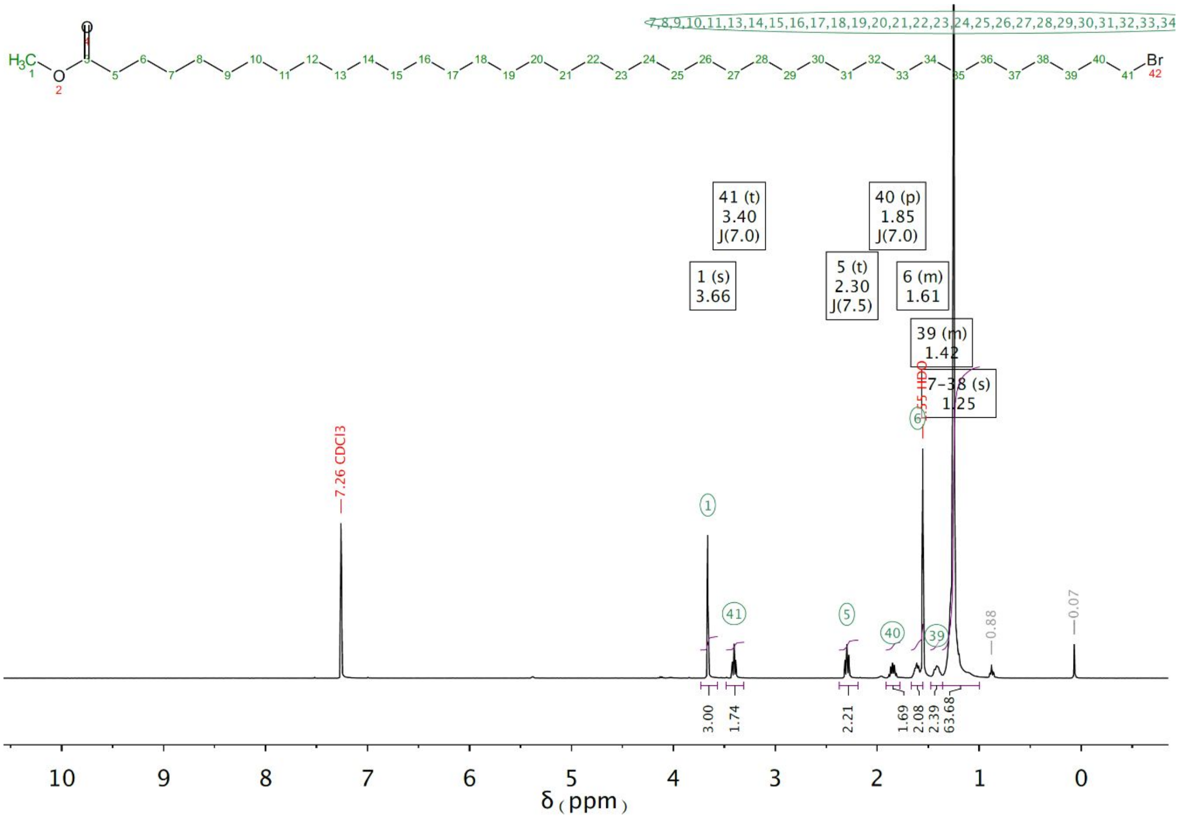

Figure S27. ^{1}H NMR (400 MHz) spectrum of **Br37ME (sat.)** in $CDCl_3$ at 298 K.

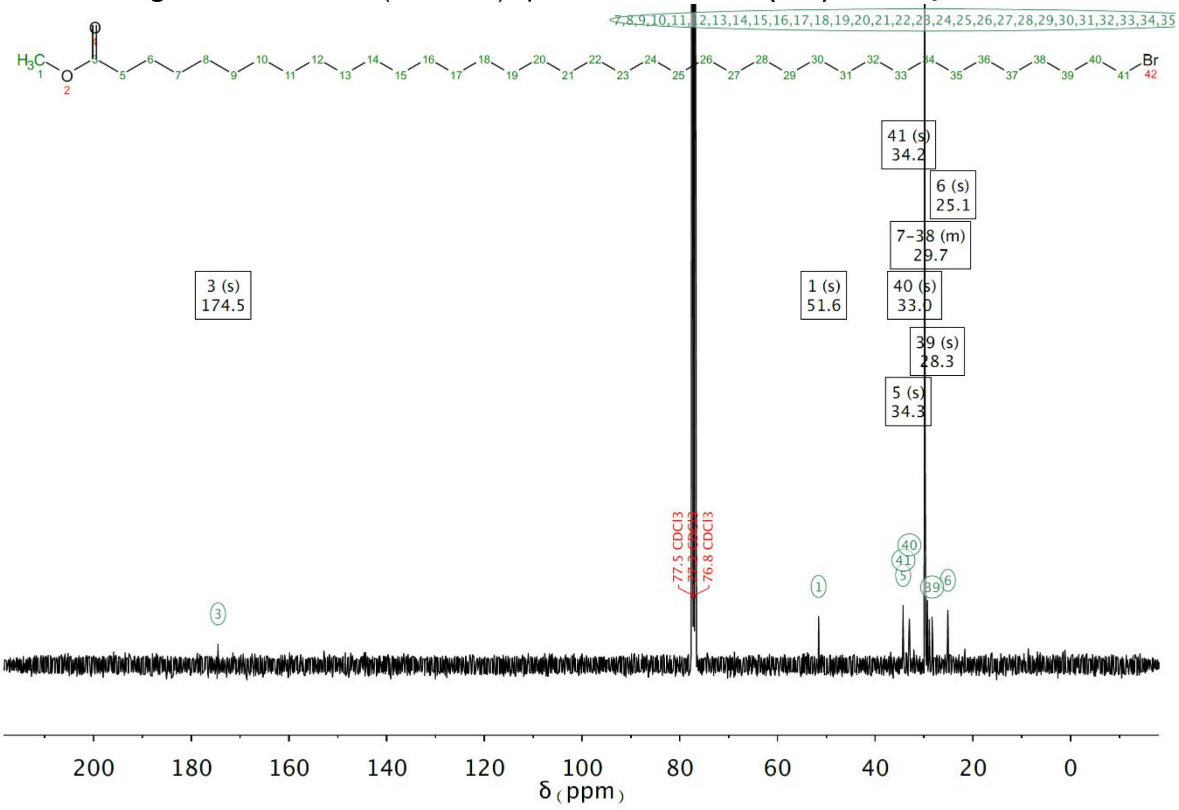

Figure S28. ^{13}C NMR (101 MHz) spectrum of **Br37ME (sat.)** in $CDCl_3$ at 298 K.

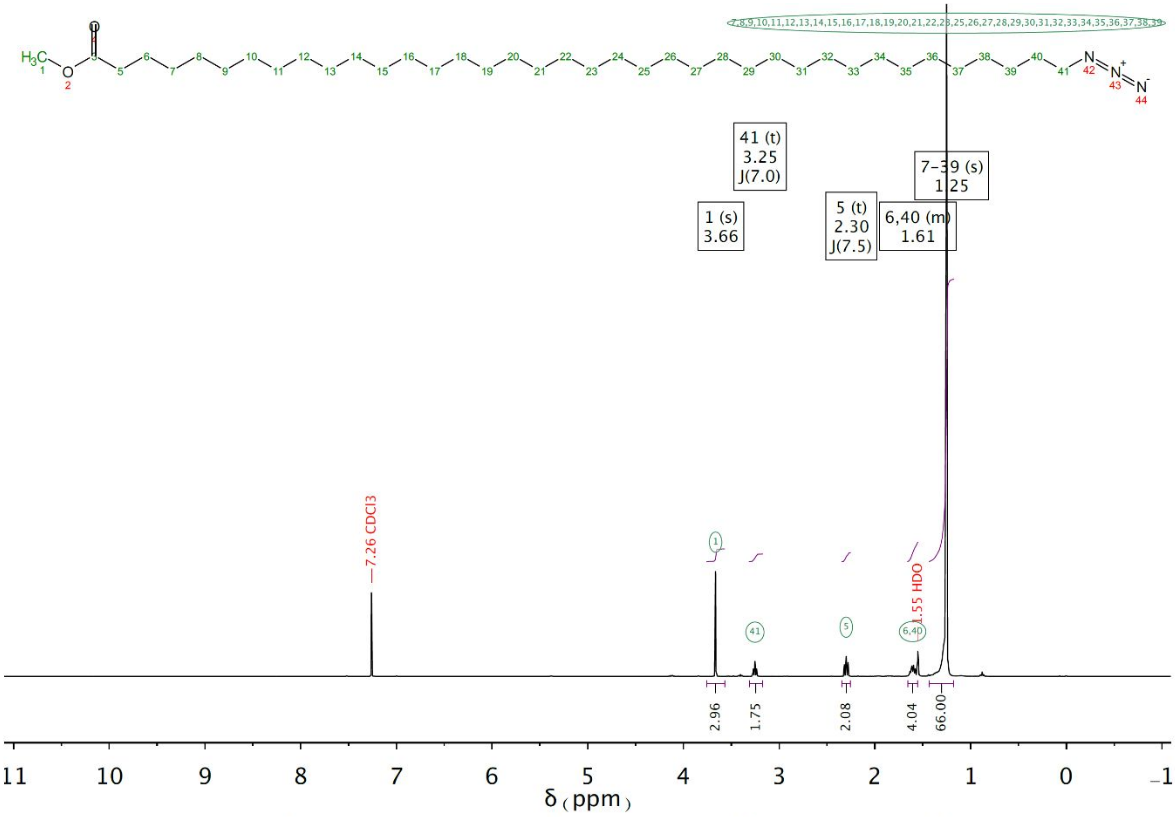

Figure S29. ^{1}H NMR (400 MHz) spectrum of **az37ME** in $CDCl_3$ at 298 K.

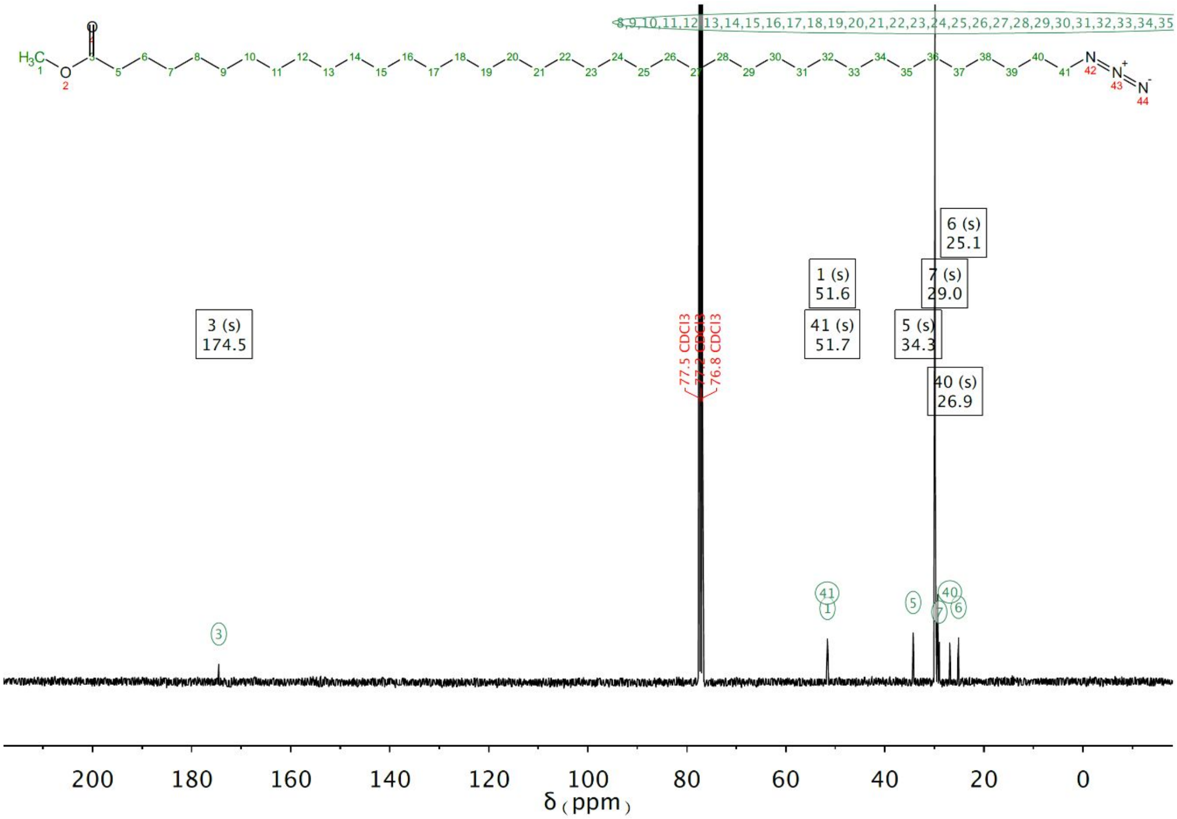

Figure S230. ^{13}C NMR (101 MHz) spectrum of **az37ME** in $CDCl_3$ at 298 K.

S2. Additional modeling data

Figure S31A shows computed waiting time dependences for the whole range of chain lengths. Thick green lines show the fits with an asymmetric double–sigmoidal function in the vicinity of the maximum. Figure S31B illustrates that the computed T_{max} values are not monotonic with the chain length caused by a small density of states of a chain band that start contributing at specific chain lengths. The W and Ro bands show significant T_{max} fluctuations at n ~ 12 (W) and 25 (Ro, Figure S31B). In an attempt to reduce the fluctuations, we introduced inhomogeneity for the tag-group frequency with respect to the chain state site frequency by averaging the dynamics for five tag frequency implementations within inhomogeneity of 12 cm^{-1}. Incorporation of the inhomogeneity led to smoother waiting-time dependences, especially for shorter chains, but could not eliminate the "noise" associated with the low chain-state density effect (Fig. S31). Note that larger inhomogeneity values were tested as well but did not reduce the noise in the computed T_{max} values.

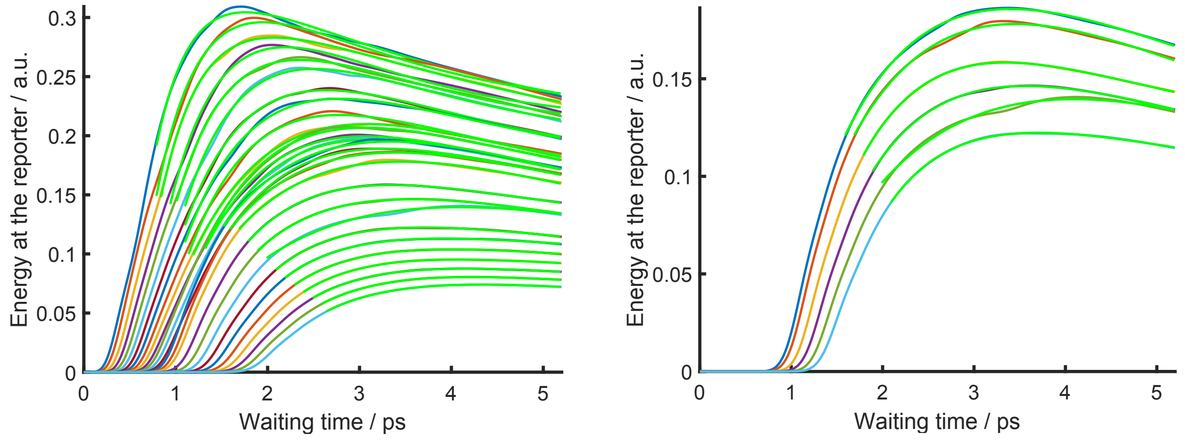

Figure S31. Computed waiting time dependences (**A**) for the whole range of chain lengths of n = 3, 4, 5, 6, 7, 8, 9, 10, 11, 12, 13, 14, 15, 16, 17, 18, 19, 21, 23, 25, 27, 29, 31, 33, 35, 37, 39 and (**B**) for six selected lengths: n = 18, 19, 21, 23, 25, and 27. The rising portion of the curves in panel **B** depends monotonically on the chain length. Thick green lines show the fits with an asymmetric double sigmoidal function in the vicinity of the maximum, $y = y_0 + A\left(\frac{1}{1+e^{-(x-xc+\frac{w1}{2})/w2}}\right)\left(1 - \frac{1}{1+e^{-(x-xc+\frac{w1}{2})/w3}}\right)$, performed in Origin software.

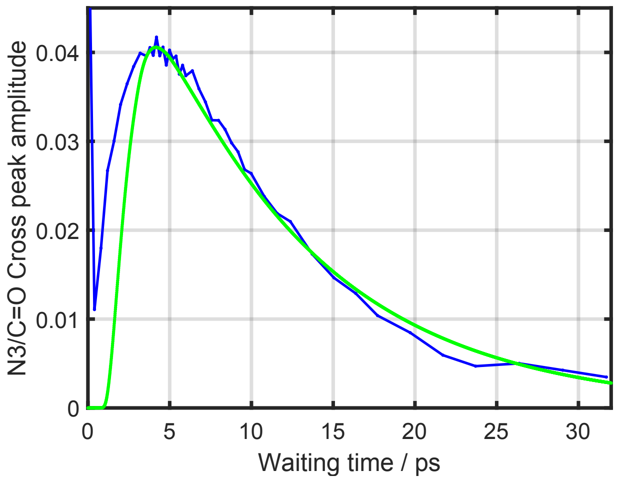

Figure S32. Experimental (blue line) and computed (green line) and waiting time dependences for *n* = 19.

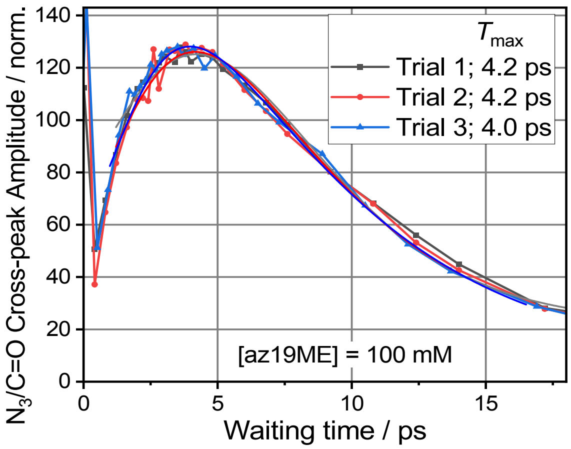

Figure S33. Waiting time traces for the $\nu_{N\equiv N}/\nu_{C=O}$ cross-peak for az19ME at 100 mM concentration. The fit curves with an asymmetric double–sigmoidal function are shown with matching colors and the obtained T_{max} values are shown as inset.

In addition to the data shown in Fig. 4 of the main text, measurements for az19ME at concentrations of 100 mM (T_{max} = 4.14 ± 0.13 ps, see Fig. S33) and 25 mM (T_{max} = 4.20 ± 0.1 ps) were performed. The resulting T_{max} values are 4.14 ± 0.13 ps (100 mM) and 4.20 ± 0.1 ps (25 mM), which match the values for 30 mM concentration of 4.20 ± 0.14 ps. Therefore, measurements at different concentrations of the guest resulted in the same T_{max} values.

References

(1) Xie, Y.; Peng, C.; Gao, Y.; Liu, X.; Liu, T.; Joy, A. Mannose-based graft polyesters with tunable binding affinity to concanavalin A. *Journal of Polymer Science Part A: Polymer Chemistry* **2017,** *55* (23), 3908.

(2) Gonzalez, C.; Sanchez, A.; Collins, J.; Lisova, K.; Lee, J. T.; Michael van Dam, R.; Alejandro Barbieri, M.; Ramachandran, C.; Wnuk, S. F. The 4-N-acyl and 4-N-alkyl gemcitabine analogues

with silicon-fluoride-acceptor: Application to 18F-Radiolabeling. *European Journal of Medicinal Chemistry* **2018,** *148*, 314.
(3) Hang, H. C.; Geutjes, E.-J.; Grotenbreg, G.; Pollington, A. M.; Bijlmakers, M. J.; Ploegh, H. L. Chemical Probes for the Rapid Detection of Fatty-Acylated Proteins in Mammalian Cells. *Journal of the American Chemical Society* **2007,** *129* (10), 2744.
